# The metagenomic data life-cycle: standards and best practices

**DOI:** 10.1093/gigascience/gix047

**Published:** 2017-06-16

**Authors:** Petra ten Hoopen, Robert D. Finn, Lars Ailo Bongo, Erwan Corre, Bruno Fosso, Folker Meyer, Alex Mitchell, Eric Pelletier, Graziano Pesole, Monica Santamaria, Nils Peder Willassen, Guy Cochrane

**Affiliations:** 1 European Molecular Biology Laboratory, European Bioinformatics Institute, Wellcome Genome Campus, Hinxton, Cambridge CB10 1SD, United Kingdom; 2 UiT The Arctic University of Norway, Tromsø N-9037, Norway; 3 CNRS-UPMC, FR 2424, Station Biologique, Roscoff 29680, France; 4 Institute of Biomembranes, Bioenergetics and Molecular Biotechnologies, CNR, Bari 70126, Italy; 5 Argonne National Laboratory, Argonne IL 60439, USA; 6 Genoscope, CEA, Évry 91000, France; 7 CNRS/UMR-8030, Évry 91000, France; 8 Université Évry val d’Essonne, Évry 91000, France; 9 Department of Biosciences, Biotechnologies and Biopharmaceutics, University of Bari “A. Moro,” Bari 70126, Italy

**Keywords:** metagenomics, metadata, standard, best practice, sampling, sequencing, data analysis

## Abstract

Metagenomics data analyses from independent studies can only be compared if the analysis workflows are described in a harmonized way. In this overview, we have mapped the landscape of data standards available for the description of essential steps in metagenomics: (i) material sampling, (ii) material sequencing, (iii) data analysis, and (iv) data archiving and publishing. Taking examples from marine research, we summarize essential variables used to describe material sampling processes and sequencing procedures in a metagenomics experiment. These aspects of metagenomics dataset generation have been to some extent addressed by the scientific community, but greater awareness and adoption is still needed. We emphasize the lack of standards relating to reporting how metagenomics datasets are analysed and how the metagenomics data analysis outputs should be archived and published. We propose best practice as a foundation for a community standard to enable reproducibility and better sharing of metagenomics datasets, leading ultimately to greater metagenomics data reuse and repurposing.

## Background

**Figure 1:**
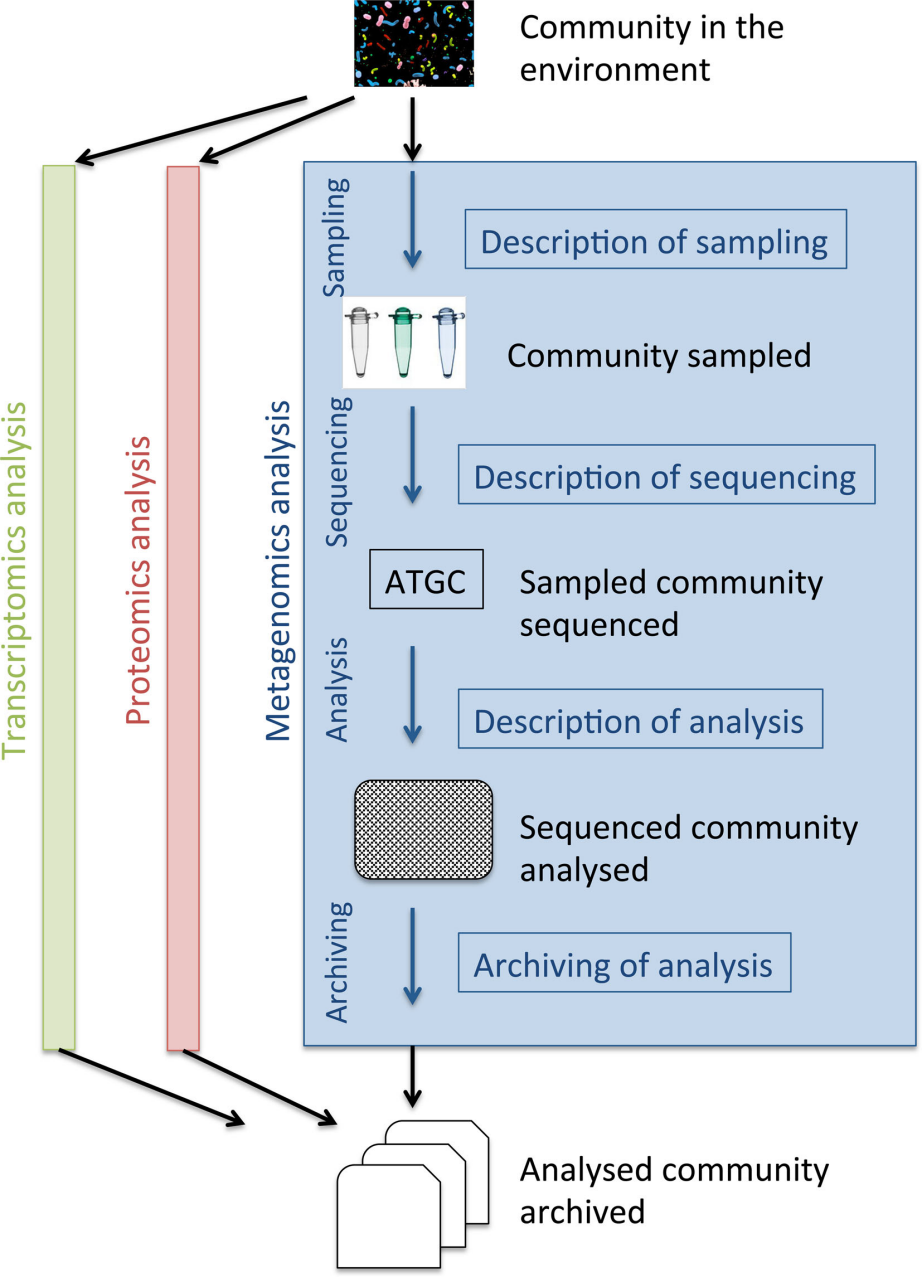
A generalized metagenomics data analysis workflow in the context of other “omics” approaches.

Recent technological advances allow researchers to examine communities of organisms using such methods as metagenomics (enumerating and exploring the genes within a community), metatranscriptomics (profiling and quantifying patterns of gene expression within a community), metabarcoding (profiling marker loci for species diversity and phylogenetic purposes), and metaproteomics (profiling the protein component of a community), enabling comprehensive insights into community composition and function (Fig. 1). The increased popularity of these meta-omics methods, driven not least by ever-decreasing cost, leads to increasing scale and complexity of experimental data and approaches to their analysis. In addition, there is growing demand for comparisons between communities that have been studied independently, often using very different approaches. However, meaningful interpretation across studies (either through aggregation and interpretation of existing published analyses or through meta-analysis of published experimental data using a uniform method) is challenging. A number of reasons exist for this: (i) each “omic” analysis workflow is a complex process, consisting of disparate and diverse tasks, ranging from sample collection and processing to data generation and analysis, where each task has many parameters that can affect analysis outputs (e.g., it has been shown that a major factor explaining correlations within metagenomics datasets can be DNA preparation and sequencing) [1]; (ii) each variable is frequently recorded in a nonstandardized way, or not recorded at all; (iii) presentation formats of the produced omics data are not unified; (iv) omics experimental data and related analysis outputs are either dispersed in several public repositories or not archived at all.

Here, we review the workflow for metagenomics data generation and analysis. Where possible, we specify essential parameters in the workflow and advise on standardized systematic reporting of these as variables. We build on the expertise of major public genomic and metagenomic resources: the European Nucleotide Archive (ENA) [2] and European Molecular Biology Laboratory (EMBL)–European Bioinformatics Institute Metagenomics (EMG) [3] at the EMBL European Bioinformatics Institute in the UK; Metagenomic Rapid Annotations using Subsystems Technology (MG-RAST) [4] at the Argonne National Laboratory in the USA; and the extensive knowledge bases in metagenomics available at research centers of excellence, the Universitetet i Tromsø in Norway, Genoscope in France, SB-Roscoff in France, and Consiglio Nazionale delle Ricerche in Italy.

For the purposes of this paper, we will predominately use marine metagenomics as a “use case” to highlight the standards environment that we describe. However, we believe that these examples will broadly translate to all areas of metagenomics research, regardless of the environment under study. From the outset, we stress that we do not wish to promote a specific workflow, but rather to demonstrate the importance of having systematic reporting conventions that accurately describe any chosen workflow, from sampling through to the presentation of analysis outputs. Our aim is to describe conventions and standards that are inclusive, extensible, and able to cope with evolving scientific developments in the field. Furthermore, where a given standard has not emerged, we will point to, or propose, a generalized “best practice” that can be used in its place. While this may produce a foundation from which a new standard could be proposed, any additional formal scientific standards need to come from the community and be ratified by scientific bodies, such as the Genomics Standards Consortium (GSC) [5].

For this paper, we have chosen a structure in which we introduce the generic data model that has been adopted by those working with metagenomic data and then move through the various practical steps—from sampling, through assay and analysis, to the archiving of analysis outputs—that a metagenomicist takes through a metagenomics investigation (see also Fig. 1).

## Overview of the Metagenomics Data Model

**Figure 2:**
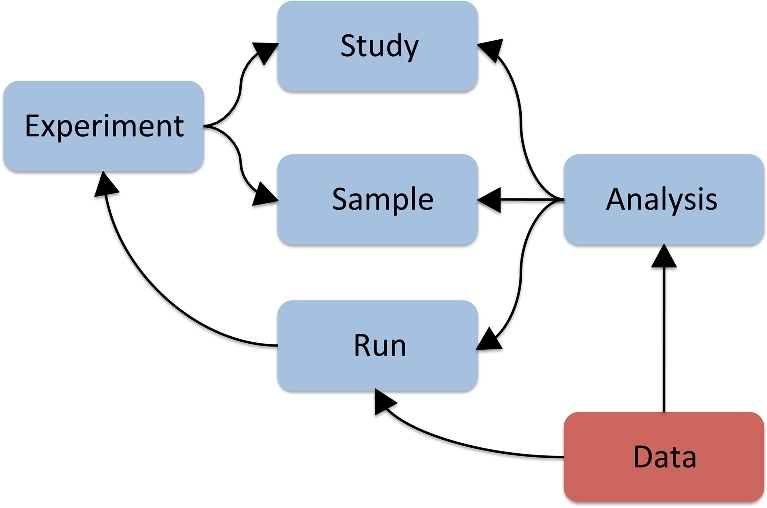
A common data model for read data and associated metadata.

The introduction of new generation sequencing technologies has enabled even small research groups to generate large-scale sequencing data. The resultant DNA sequences and associated information are typically captured in several interconnected objects (Fig. 2), which represent the following concepts:

- Study: information about the scope of a sequencing effort that groups together all data of the project;
- Sample: information about provenance and characteristics of the sequenced samples.
- experiment: information about the sequencing experiments, including library and instrument details;
- Run: an output of a sequencing experiment containing sequencing reads represented in data files;
- Analysis: a set of outputs computed from primary sequencing results, including sequence assemblies and functional and taxonomic annotations.

Information associated with DNA sequence is frequently referred to as “metadata.” This includes all information described in the Study, Sample, Experiment, and Run data objects, spanning sampling context, description of sample processing, experimental design, library creation, sequencer configuration, and provenance information required for attribution and credit to comply with best scientific practice for publication in the academic literature and to inform processes around Access and Benefit Sharing. Primary data represent, in this context, primary “raw” experimental sequence reads produced by sequencing machines. (On occasion, some basic data processing, such as quality control (filtering out of poor-quality reads, clipping of low-quality regions, etc.), is applied to “raw” primary data, and these processed data are retained as primary; while it is preferable to retain true “raw” primary data, perhaps in addition to these processed data, it is important to apply broadly accepted processing methods and to describe these methods as part of the metadata record.) Following this, for some metagenomics studies, the primary data are analysed directly (e.g., 16S or 18S rRNA gene amplicon studies), while in others, they are assembled into contigs before undergoing further analysis. Regardless of the approach, the output of any computational analysis process (including assembly) on the primary data is here referred to as derived data. We discuss derived data in more detail below, but the more harmonized the formats and validations for data and metadata objects, the more easily the generated data can be shared, discovered, re-used, and repurposed.

Each metagenomics initiative has a scope, aim, and 1 or more (human) contributors in each step of the workflow, who may be distributed over a wide geographical area. It is essential to capture contextual information regarding the contributors as this supports appropriate attribution and credit and clarifies the responsible parties for each step of the workflow. Contributors to (i) material sampling, (ii) primary data generation, and (iii) derived data generation should always be clearly presented in data records. Minimum metadata checklists frequently do not specifically capture data generating or contributing institutions. However, this information is frequently available and can be parsed from the registration systems for reporting individual steps of the data generation workflow or from associated peer-reviewed publications.

## Sampling

The method of collecting a sample (a fundamental unit of material isolated from the surrounding environment) is dictated by the nature of the community under investigation, the environment in which it is found, and the type of “omics” investigation being performed. The slightest deviation in method, regardless of the protocol chosen, can have a profound impact on the final “omics” analysis results. It is therefore essential that the details of the sampling process are captured accurately and in a standardized way.

Domain experts are in the best position to formulate opinions on the general scope and content of contextual data (environmental characteristics observed or measured during sample collection) and methodological variables (such as sampling volume and filtration method). These opinions are conventionally formalized as data reporting standards by community initiatives such as the GSC for genomics data [5]—on several of which we expand below—the Proteomics Standards Initiative [6] for proteomics data, or the Group on Earth Observations Biodiversity Observation Network for the various dimensions of biodiversity, including genetic variation and biodiversity data [7, 8].

The Minimum Information about Metagenomic Sequence (MIMS) [9] is a GSC-developed data reporting standard designed for accurate reporting of contextual information for samples associated with metagenomic sequencing, and it is also largely applicable to metatranscriptomics studies. Minimum Information about a MARKer gene Sequence (MIMARKS) [10] is another GSC-developed contextual data reporting standard for reporting information about a metabarcoding study, which is referred to in the standard as the “MIMARKS-survey investigation type.”

MIMS and MIMARKS are a part of a broader GSC standard, the Minimum Information of any (x) Sequence (MIxS) [11], which describes 15 different environmental packages that can be used to specify the environmental context of a sequenced microbial community, such as air, water, or host organism-associated. The MIxS descriptors can be combined with any environmental package and together provide rich information on sampling context.

To illustrate, Table 1 summarizes the minimum set of elements required for description of a metagenomic sample taken from an aquatic environment. It uses MIMS mandatory descriptors, combined with the mandatory descriptors of the Water Environment package. Similarly, a sample taken from the gut of a fish host can be described using the MIMS core descriptors, in combination with descriptors in the host-associated Environment package. The 15 different environmental packages defined by the GSC are available from the GSC website as a single bundled download [12] and are presented in a host of informatics tools that support data reporting and presentation, such as the submission tools of the databases of the International Nucleotide Sequence Database Collaboration [13] and ISAtools [14]. It remains up to the experimentalist to choose the most appropriate package from within the checklist bundle for their study, thereby defining the list of fields that will be used to capture relevant metadata. Before embarking on a metagenomics study, we recommend that the appropriate checklist be identified, so that the appropriate metadata can be captured during the experiment, rather than retrospectively having to determine these metadata.

**Table 1:** Checklist of MIMS mandatory descriptors for a sample taken from an aquatic environment and associated with a metagenomic sequencing experiment.

MIMS-mandatory water sample provenance descriptors	Descriptor format
Submitted to INSDC	Boolean
Project name	Text
Investigation type	Fixed value: “metagenome”
Geographic location (latitude and longitude)	Decimal degrees in WGS84 system
Depth	Metres: positive below the sea surface
Geographic location (country and/or sea region)	INSDC country list [51]
Collection date	ISO8601 date and time
Environment (biome)	ENVO class [52]
Environment (feature)	ENVO class
Environment (material)	ENVO class
Environment package	MIxS controlled vocabulary [12]

ENVO: Environment Ontology.

The Marine Microbial Biodiversity Bioinformatics and Biotechnology (M2B3) data reporting and service standard [15] specifically addresses contextual data relating to marine microbial samples. It represents a common denominator of contextual data from data standards used in the Public Genomic Data Archives (MIxS, version 4.0) [12], Pan-European Network of Oceanographic Data Archives (Common Data Index schema, version 3.0) [16], and Pan-European Network of Biodiversity Data Resources (Ocean Biogeographic Information System schema, version 1.1) [17]. This M2B3 unified data standard significantly simplifies contextual data reporting as it provides an interoperable solution for sharing contextual data across data archives from different scientific domains. A minimum M2B3 checklist for reporting contextual data associated with marine microbial samples is summarized in Table 2.

**Table 2:** Checklist of M2B3 mandatory descriptors for a microbial sample taken from a saline water environment and associated with a metagenomic sequencing experiment.

M2B3-mandatory saline water sample provenance descriptors	Descriptor format
INVESTIGATION_campaign	Text
INVESTIGATION_site	Text
INVESTIGATION_platform	SDN: L06 controlled vocabulary [53]
EVENT_latitude	Decimal degrees in WGS84 system
EVENT_longitude	Decimal degrees in WGS84 system
EVENT_date/time	ISO8601 date and time in UTC
SAMPLE_title	Text
SAMPLE_protocol label	Text
SAMPLE_depth	Metres; positive below the sea surface
ENVIRONMENT_environment (biome)	ENVO class
ENVIRONMENT_environment (feature)	ENVO class
ENVIRONMENT_environment (material)	ENVO class
ENVIRONMENT_temperature	SDN: P02 [54], SDN: P06 [55] controlled vocab.
ENVIRONMENT_salinity	SDN: P02, SDN: P06 controlled vocab.

ENVO: Environment Ontology; SDN: SeaDataNet; UTC: coordinated universal time.

For most adopted standards of this type, only a few fields of contextual data are mandatory, reflecting the balance between usability for the experimentalist reporting his/her science and consumers re-using this science; limiting the number of mandatory fields lowers the burden for experimentalists to comply with the standard, while a small number of parameters are universally, or near-universally, required for downstream analysis. The importance of the optional MIxS and M2B3 fields for metagenomic data analysis is detailed in Table 3.

**Table 3:** Selection of nonmandatory MIxS and M2B3 descriptors (column B) and formats (column D).

A: group	B: nonmandatory sample provenance descriptors	C: standard	D: descriptor format	E: value for analysis (H/M/L)
1	Sample collection device or method	MIxS (MIMS)	Text	H
1	EVENT_device	M2B3	Text	H
1	EVENT_method	M2B3	Text	H
2	Sample material processing	MIxS (MIMS)	Text	H
3	Amount or size of sample collected	MIxS (MIMS)	Numeric & unit	H
3	SAMPLE_quantity (e.g., length, mass)	M2B3	Text	H
4	Sample storage location	MIxS (water)	Text	L
4	SAMPLE_container (e.g., storage container)	M2B3	Text	L
5	Sample storage duration	MIxS (water)	Interval	H
6	Sample storage temperature	MIxS (water)	Numeric & unit	H
6	SAMPLE_treatment_storage (e.g., temperature)	M2B3	Text	H
7	Chemical administration	MIxS (water)	CHEBI ontology [56]	M
7	SAMPLE_treatment_chemicals	M2B3	CHEBI ontology	M
8	SAMPLE_size_fraction_upper_threshold	M2B3	Text	H
8	SAMPLE_size_fraction_lower_threshold	M2B3	Text	H
9	SAMPLE_content (e.g., 0.22 μm filter, 20mL water)	M2B3	Text	H
10	Concentration of chlorophyll	MIxS (water)	Numeric & unit	HM
10	ENVIRONMENT_ecosystem_pigment concentration	M2B3	SDN: P02, SDN: P06 controlled vocab.	HM
11	Fluorescence	MIxS (water)	Numeric & unit	HM
11	ENVIRONMENT_ecosystem_fluorescence	M2B3	SDN: P02, SDN: P06 controlled vocab.	HM
12	Density	MIxS (water)	Numeric & unit	M
13	Organism count	MIxS (water)	Numeric & unit	ML
13	ENVIRONMENT_ecosystem_picoplankton (flow cytometry) abundance	M2B3	SDN: P02, SDN: P06 controlled vocab.	ML
13	ENVIRONMENT_ecosystem_nano/microplankton abundance	M2B3	SDN: P02, SDN: P06 controlled vocab.	ML
13	ENVIRONMENT_ecosystem_meso/macroplankton abundance	M2B3	SDN: P02, SDN: P06 controlled vocab.	ML
14	Primary production	MIxS (water)	Numeric & unit	M
14	ENVIRONMENT_ecosystem_primary production	M2B3	SDN: P02, SDN: P06 controlled vocab.	M
15	Bacterial production	MIxS (water)	Numeric & unit	M
15	ENVIRONMENT_ecosystem_bacterial production	M2B3	SDN: P02, SDN: P06 controlled vocab.	M
16	Biomass	MIxS (water)	Numeric & unit	ML
16	ORGANISM_biomass	M2B3	Numeric & unit & method	ML
17	ORGANISM_biovolume	M2B3	Numeric & unit & method	L
18	ORGANISM_size	M2B3	Numeric & unit & method	L
19	INVESTIGATION_authors	M2B3	Text	M
20	Host taxid	MIxS (host associated)	NCBI taxonomy identifier [57]	M

These descriptors cover such areas as the structure or viability of the community under investigation and sample pooling procedures. Column A groups descriptors that are related conceptually (1 – sample collection method & device, 2 – sample processing, 3 – sample quantity, 4 – storage container, 5 – storage duration, 6 – storage temperature, 7 – chemical treatment, 8 – microbial fraction thresholds, 9 – sample content, 10 – pigment concentration, 11 – fluorescence, 12 – density, 13 – organism abundance, 14 – primary production, 15 – bacterial production, 16 – organism biomass, 17 – organism biovolume, 18 – organism size, 19 – investigation contributors, 20 – unique taxonomic index identifier for organism host). Column C shows the descriptor association with the respective contextual data reporting the standard suitable for marine metagenomic data. Column E suggests the descriptor's importance for metagenomic data analysis (H – high relevance, M – medium relevance, L – low relevance).

CHEBI: Chemical Entities of Biological Interest; SDN: SeaDataNet.

We wish here to note a convention on the handling of replicate samples. Since biological replicates are separate physical entities, we recommend that multiple sample records are registered, 1 for each biological replicate, with reciprocal references represented as a sample attribute with the name “biological replicate” and attribute value provided as the accession number(s) of the related biological sample(s). In contrast, “technical replicates,” for which only a single sample exists, are treated downstream in the workflow.

Consistent and rich contextual data can become a powerful tool for metagenomics data analysis. Two marine studies, the *TARA* Oceans sequencing study (PRJEB402) [18] and Ocean Sampling Day (OSD; PRJEB5129) [19] both use the same M2B3 contextual data reporting standard, enabling comparison of data within and across studies. For instance, data from the *TARA* Oceans shotgun sequencing of the prokaryotic fraction filtered from seawater (PRJEB1787) [20] can be compared to the shotgun data from OSD (PRJEB8682) [21], enabling detailed or complex queries. Specifically, a taxonomic or functional profile from the *TARA* Oceans sample from a depth of 5 m and salinity of 38 psu (SAMEA2591084) [22] can be compared to profiles of the OSD sample from a depth of 5 m (SAMEA3275502) [23] or the OSD sample with the same salinity of 38 psu (SAMEA3275531) [24]. In contrast, very few conclusions can be drawn from a comparison to a sample with insufficient contextual information (SAMN00194025) [25].

Details of the project investigators are usually recorded in the Study metadata object, and sampling contextual data are mostly captured in the Sample metadata object (Fig. 2). A common way to standardize reporting of contextual data is via a checklist of key value pairs, thereby ensuring that parameters of a similar kind are described consistently. Furthermore, syntactic and semantic rules can be predefined in the checklist, enabling validation of compliance with these rules. For instance, automated checks can be applied to test whether a mandatory descriptor (key) in the checklist has a value and whether the value is in a specified format. Each element to be checked can be predefined as text, a class or term from an ontology, a controlled vocabulary or taxonomic index, or formulated as a regular expression. (Regular expressions can be used, e.g., to check that the key “collection date and time” complies with International Standards Organisation's [ISO’s] 8601 standards and that numeric values lie within a defined range.)

The most common formats for sharing Study and Sample metadata are Extensible Markup Language (XML), tab-separated values, ISA-tab, or JavaScript Object Notation formats. Examples of the Study and Sample XML are available from the European Nucleotide Archive [26, 27], where the files are also validated against the XML schema [28]. Regardless of the format used to supply the metadata, because they all use the same underlying standards, a simple translation between the formats enables different data to be compared. This allows scientists to use the different tools or approaches that they are most familiar with, whilst ensuring consistent delivery of the metadata.

## Sequencing

Once a sample is collected and its provenance recorded, it is subjected to preparation steps for nucleotide sequence analysis. This may happen immediately after sampling or in stages over many months. Processing steps cover all handling of the sample leading to the DNA isolation. Although MIxS covers some of the metadata fields for reporting the DNA extraction steps, it is extremely difficult to define a generic set of fields describing the DNA extraction method with a high granularity due to its complexity and diversity. For example, it might be relatively straightforward to identify variables for reporting isolation of DNA from a seawater sample, but that will not suit the more complex DNA isolation procedure for a sediment sample. We suggest the best practice here is to use the existing MIxS fields, such as sample material processing, nucleic acid extraction, and nucleic acid amplification, for concise description of the nucleic acid preparation. A detailed description, or a reference to the material preparation steps recorded in a data resource that specializes in protocol capture and dissemination, such as protocols.io [29], is important due to the significant influence this can have on the observed profile of the microbial community under investigation.

Equally critical for the downstream metagenomic data analysis and interpretation is the reporting of sequencing library preparation protocols and parameters as well as sequencing machine configurations.

Table 4 shows mandatory descriptors for new generation nucleotide sequencing experiments as currently captured by International Nucleotide Sequence Database Collaboration (INSDC) databases. Table 5 lists nonmandatory descriptors including MIMS sequence-related descriptors and provides our opinion on the importance of these descriptors for metagenomic data analysis. Note that while a number of controlled vocabularies have been developed for accurate recording of sequencing experiment parameters, the evolution of these constrained vocabularies is very dynamic and driven by technological advances.

**Table 4:** Mandatory descriptors for sequencing.

Mandatory descriptors of sequencing provenance	Descriptor format
Instrument platform	Controlled vocabulary [Illumina, Oxford Nanopore, PacBio smrt, Ion Torrent, ls454, Complete Genomics, Capillary]
Instrument model	Controlled vocabulary [58]
Library source	Controlled vocabulary [59]
Library strategy	Controlled vocabulary [60]
Library selection	Controlled vocabulary [61]
Library layout	Controlled vocabulary [single, paired]
Read file name	Text
Read file md5 checksum	32-digit hexadecimal number
Second read file name (for paired Fastq files)	Text
Second read file md5 checksum (for paired Fastq files)	32-digit hexadecimal number

**Table 5:** Nonmandatory sequencing descriptors (column A) and formats (column B); column C suggests the descriptor's potential importance for metagenomic data analysis (H – high relevance, M – medium relevance, L – low relevance).

A: nonmandatory descriptors of sequencing provenance	B: descriptor format	C: value for analysis (H/M/L)
Sequencing centre contact	Text	M
Sequencing experiment name	Text	L
Library name	Text	L
Library description	Text	L
Library construction protocol	Text	M
Library construction method (MIMS)	Text	M
Library size (MIMS)	Numeric	M
Library reads sequenced (MIMS)	Numeric	M
Library vector (MIMS)	Text	M
Library screening strategy (MIMS)	Text	M
Insert size (for paired read files)	Numeric	M
Spot layout (for SFF read files)	Controlled vocabulary (single, paired FF, paired FR)	M
Linker sequence (for SFF read files)	Sequence of nucleotides	H
Multiplex identifiers (MIMS)	Sequence of nucleotides	H
Adapters (MIMS)	Sequence of nucleotides	H
Quality scoring system (for Fastq files)	Controlled vocabulary (phred, log-odds)	H
Quality encoding (for Fastq files)	Controlled vocabulary (ASCII, decimal, hexadecimal)	H
ASCII offset (for Fastq files)	Controlled vocabulary (!, @)	H
Nucleic acid extraction SOP (MIMS)	Text	H
Nucleic acid amplification SOP (MIMS)	Text	H
Sequencing coverage	Numeric	H

Variable parameters of the library preparation and instrumentation are captured in the metadata objects Experiment and Run (see Fig. 2). Examples of the Experiment and Run XML are available, e.g., from the ENA [30, 31]. Each Experiment should refer to Study and Sample objects, to provide context for the sequencing, and is referred to from the Run objects, which point to the primary sequencing reads.

The primary data (the reads) are stored in files of various formats, which can be standard (Binary Alignment/Map [BAM], Compression Reduced Alignment/Map [CRAM], or Fastq) or a platform specific, as with standard flowgram format (SFF), PacBio, Oxford Nanopore, or Complete Genomics. Information on the read data format must be indicated in the description of sequencing.

The minimum information encapsulated in read data files includes base calls with quality scores. Quality requirements on read data files are file format specific and are summarized, e.g., in the ENA data submission documentation [32]. A freely available diagnostic tool for the validation of CRAM and BAM files is the Picard ValidateSamFile [33]. Validation of Fastq files is less straightforward since there is no single FASTQ specification. Recommended usage of FASTQ can be found, e.g., in the ENA guidelines [34]. An open resource for managing next generation sequencing datasets is the NGSUtils [35], which also contains tools for operations with FASTQ files. As sequencing technologies change over time, the formats and associated validation tools may well change, so a comprehensive list of formats and tools is likely to become outdated. The key point is to adopt a widely used format and to check for file format and integrity (e.g., checksums).

## Analysis

Standards in metagenomics for the description of sampling and sequencing have grown out of those from more traditional genomics. While there are still some shortcomings in these standards, as highlighted in the previous sections, metadata concerning sampling and sequencing are commonly captured for metagenomics studies. Compliance is high partly due to the scientific journals requiring scientists to submit sequence data to an INSDC database prior to publication. However, there are currently no standards for reporting how metagenomics datasets have been analysed. While systematic analysis workflows, such as those offered by EMG, Integrated Microbial Genomes with Microbiomes [36], META-pipe [37], and MG-RAST, provide a standard that is documented (albeit in different ways), many published datasets are analysed by in-house bespoke pipelines. Although many authors provide an outline in the “materials and methods” or “supplementary materials” section of their publications, it is rarely possible to reproduce the analysis from this alone, due to missing software parameters, lack of detail on software versions, and ambiguous reference databases and their associated versions.

Typically, once the sequence read files have been produced, they are analysed using 1 or more workflows [38], with each workflow comprising different data processing or analysis components. Most workflows involve aspects such as quality control (e.g., removing sequences that fail to meet predefined quality scores), assembly, sequence binning (e.g., identifying 16S rRNA genes or protein coding sequences), and taxonomic classification of sequences and/or functional prediction. However, each workflow will be tailored to how the sample has been processed and the question being addressed. For example, if a sample has been size-fractioned for viruses using a 0.22 μm filter, there would be little point analysing the data for eukaryotic 18S rRNA as any eukaryotic organisms would have been physically removed from the sample before the DNA extraction process.

Analysis workflows typically have 1 or more of the following components:

1. central algorithmic software, which may be from a third-party source;
2. “glue” software that may ensure input/output formats or split/join input files for parallelization;
3. reference datasets that are used by (i). For example, the Greengenes database of 16S rRNA genes [39], SILVA database of 16S/18S small subunit ribosomal RNA genes [40], and the National Center for Biotechnology Information nonredundant database of nonidentical protein sequences [41], for taxonomic or functional analysis.

However, even knowing these elements may not be sufficient for analyses to be independently recreated. For example, the algorithm may accept a set of input parameters that can be used to fine-tune an analysis, such as selecting an E-value threshold for determining the significance of a sequence match to a reference database. Other parameters may influence speed-performance, which allows the original analysis to complete in a timely fashion, but they may or may not have an effect on the results. For example, running *hmmsearch* from the HMMER package, changing the number of central processing units (CPUs) used will not change the results, but changing options on the heuristics such at the –F1 threshold (which controls the number of sequences passing the first heuristic state) may alter the output; both will potentially increase performance in terms of speed.

Capturing and reporting all provenance information is essential to understand exactly what analysis has been performed on the data and to ensure reproducibility [42]. The use of publicly available analysis pipelines (such as EMG, MG-RAST, or META-pipe) helps with this process since analysis is performed using predefined components, settings, and databases (or, in some cases, using user-selected components, selected from a predefined list of options). Nevertheless, capturing analysis metadata remains essential as, e.g., MG-RAST allows the users to dynamically set E-value thresholds after the pipeline analysis has been performed. Furthermore, the tools, libraries, and reference databases used by the pipelines are regularly updated, and thus capturing analysis provenance information is vitally important and should be systematically “tagged” to the results.

To date, there is no universally endorsed “analysis standard” for describing and recreating a metagenomics analysis pipeline, and without this standard (and subsequent adoption/enforcement), it will continue to be difficult, if not impossible, to reproduce analysis workflows. However, all is not lost. “Workflows” and their definitions is an active field of computer science research, and potential solutions are already available, including Common Workflow Language, Yet Another Workflow Language, Business Process Execution Language, and Microsoft Azure's Workflow Definition Language, to name but a few. Several of the co-authors for this publication already participate in the GSC M5 consultation group, which aims to define a standard enabling the recreation and exchange of metagenomics datasets. In the absence of a standard, we believe it is important to define some of the basic best practices, which an accepted standard would formally encapsulate. For simplicity, we will focus on a single “best practice” use case: the description of the analysis of a run. Other types of analysis, such as pooling of runs or comparing results between runs, are beyond the scope of this article.

**Figure 3:**
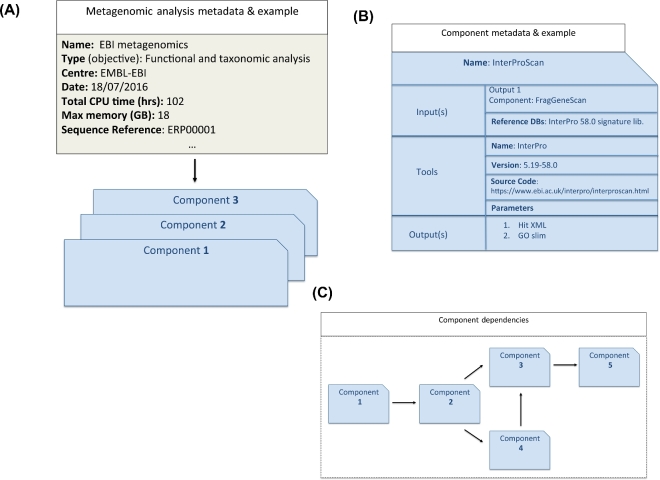
Schematic overview of best practice for analysis metadata collection with example fields. A) Overarching metadata; B) Analysis component; C) Workflow.

A schematic overview of a best practice for analysis metadata collection is shown in Fig. 3A. An overarching set of metadata relating to analysis should encapsulate generic information such as analysis centre, name of bioinformaticians, analysis objectives, name of overall analysis (if appropriate), and the date on which the analysis was performed. It should also contain appropriate pointers to the run data, run sequence metadata, and associated sample data. Underneath the overarching analysis metadata is a collection of analysis components that describe each stage of the analysis (Fig. 3B). Each component can be divided into 3 sections: input(s), analysis algorithm, and output(s).

The input section should describe the details of the various inputs to the analysis, which could be the raw sequence reads or the output of another analysis component, and reference databases and their provenance data, such as version, where necessary. The analysis section should contain the algorithm tool, version, all parameters used, and a basic description of the analysis. The output section should describe each output from the analysis, together with a description of contents and format.

Each analysis component could then be coupled to form an analysis workflow, as shown in Fig. 3C. The workflow may be in a portable intermediate format that can be submitted to a workflow manager for execution in a specific environment.

This best practice framework is merely that—a best practice, and we have not touched on the technical issues of how to capture this information or on controlled vocabularies (since these need to come from the community). Furthermore, enforcing compliance and validation against the standard will also require a community effort. Complete validation would require the standard to be machine readable and deployable, with potentially the need to have small “test” datasets and their associated results to perform regression testing of the analysis metadata. However, who is responsible for validation and what happens if something fails after publication are open questions. This could arguably be a step too far; currently sampling and sequence metadata are validated against the standard, but taken in good faith to be correct beyond this.

## Analysis Results Archiving—A Final Piece?

Having an analysis provenance standard would allow metagenomics analysis results to be recreated more readily. While this is undoubtedly an important and necessary step, it has a major limitation within the community. As indicated [43], the fraction of money spent on informatics from an overall project budget is increasing dramatically. Metagenomics datasets tend to be large, in the order of gigabyte–terabyte (TB), and processing may take thousands of CPU hours, restricting reanalysis to only those with significant compute resources. For example, the subset of the *TARA* Oceans Ocean Microbiome Project (PRJEB7988) [44] that has been size-fractioned for prokaryotes comprises 135 samples with 248 runs containing 28.8 billion reads. The analysis output represents about 10 TB of data, with 23.2 billion predicted protein coding sequences. Thus, reanalysis would be costly and potentially wasteful if a particular workflow had already been run on the data. Therefore, a final step in a metagenomics analysis is the appropriate archiving of results. There is an obvious cost-benefit balance to be drawn here as storing every intermediate of a workflow would lead to an explosion of data. Clearly, key intermediates and outputs of an analysis workflow need to be determined. These key archived components will be tailored to the analysis, but should at least include operational taxonomic unit counts and assignments, functional assignment counts, and read/sequence positional information for the aforementioned assignments. Such data files are already made available from MG-RAST and EMG, and those from other sources are accepted for archiving within ENA.

If metagenomic assemblies have been performed, then these should have an appropriate structure of contigs, scaffolds, or chromosomes with an appropriate format as detailed, e.g., in the ENA data submission documentation [45]. Due to the overheads of producing an assembly, these should be archived, ideally with an INSDC database.

The data model for metagenomics, as described in Fig. 2, represents metagenomic analysis results in the data Analysis object with appropriate pointers to the corresponding run sequence metadata and associated sample collection contextual data. While there is an established practice to archive primary sequence data in the Run object and assemblies of the primary sequences in the Analysis object, it is not a common practice to archive results of functional and taxonomic metagenomic analysis of in-house bespoke pipelines. It would be beneficial to the metagenomics community to include this in the best practice, and such data are accepted by ENA for archiving. The metagenomics standard environment reviewed here as well as outcomes of the GSC M5 consultation group can contribute to defining required descriptors of the Analysis object for archiving of metagenomics analysis results, which can serve as a framework for the exchange of metagenomics datasets on a routine basis, similarly as is currently done for the primary sequence data.

## Future

One challenge over the next several years will be the validation of compliance across the entirety of the standards and best practice that we have covered. While validation tools and recommended practices exist for parts (e.g., contextual data descriptors using MIxS-compliant validation tools from ISA and experimental descriptors upon submission to an INSDC database), not all parts have such maturity (e.g., analysis descriptors) and there exists no overarching validation protocol for an entire metagenomics study. The GSC is aiming to contribute in this area with the introduction of MIxS “profiles” to provide an overlay on top of MIxS environmental packages and the core MIxS fields. These profiles will enable the creation of tool suites for compliance checking. In addition (and perhaps more importantly), they will enable groups of researchers, institutes, funders, and other communities to define levels of compliance for contextual datasets. Examples of this are the National Science Foundation National Ecological Observatory Network [46] and the National Science Foundation Critical Zone Observatory [47] networks that are working with the GSC to establish silver, gold, and platinum sets of parameters that need to be provided and validated for datasets to be compliant. A key moment in the acceptance of said new profiles will be the availability of tool support for data creators, end-users, and portals. Imagine, e.g., a search for datasets in EMG or MG-RAST that allows restriction of the search to just platinum-level datasets. For the data consumer, this will result in better ways of telling their science story using third-party data, and for the data creator, this will provide guidance on what to create for a specific community. In addition, funding agencies can require certain minimal compliance.

A further area to be addressed is that of standards around descriptions of metagenomics analyses. The creation of a lightweight data standard for an Analysis object that allows easy transfer of analyses is a key goal of the GSC M5 initiative, but the complexity of the task and lack of dedicated resourcing has rendered progress slow; while frameworks and systems for recording analysis provenance need to be established, we have aimed to indicate in this publication a set of indications for best practice that can form the foundation for a community standard enabling the recreation and exchange of metagenomics datasets. Improving standardization will also help raise clarity in the literature around metagenomics through a tightening of language. For example, Ultrafast Protein Classification [48] uses Pfam [49] matches as a reference library, with the results being referred to as a “Pfam hit.” However, this may not necessarily be a Pfam hit as a Pfam hit is defined as a sequence match scoring greater than the Pfam-defined threshold to the Pfam profile Hidden Markov Model (i.e., the Pfam database method).

## Conclusions

In this overview of the metagenomics standard environment, we have outlined best practice for the reporting of metagenomics workflows. We have reviewed the essential steps: (i) material sampling, (ii) material sequencing, (iii) data analysis, and (iv) data archiving, and highlighted essential variable parameters and common data formats in each step.

Reporting on the provenance of a sample and associated nucleotide sequence data is largely established by public sequence data repositories and is also being addressed by contextual data standardization initiatives. In contrast, a reporting standard on metagenomics data analysis is absent, yet the high complexity of metagenomics creates a pressing demand for establishing such a practice. Capturing key metadata relating to analysis would greatly improve reproducibility. Archiving key results of metagenomics data analysis would allow a more accurate evaluation of the benefits of reproducing the analysis.

Only by adopting these standards and best practices can metagenomics data be assessed against the Findable, Accessible, Interoperable, and Reusable (FAIR) principles that should be applied to any scientific dataset [50].

## Abbreviations

BAM: Binary Alignment/Map; CPU: central processing unit; CRAM: Compression Reduced Alignment/Map; EMBL: European Molecular Biology Laboratory; EMG: EBI Metagenomics; ENA: European Nucleotide Archive; ENVO: Environment Ontology; GSC: Genomic Standards Consortium; GSC M5: Genomic Standards Consortium M5 working group; INSDC: International Nucleotide Sequence Database Collaboration; ISO: International Standards Organisation; MG-RAST: Metagenomic Rapid Annotations using Subsystems Technology; MIMS: Minimum Information about a Metagenome Sequence; MIMARKS: Minimum Information about a MARKer gene Sequence; MIxS: Minimum Information about any (x) Sequence; M2B3: Marine Microbial Biodiversity Bioinformatics and Biotechnology; OSD: Ocean Sampling Day; SDN: SeaDataNet; SFF: standard flowgram format; TB: terabyte; XML: Extensible Markup Language.

## Funding

This work was supported by the ELIXIR-EXCELERATE, funded by the European Commission within the Research Infrastructures programme of Horizon 2020, grant agreement number 676 559.

## Competing interests

The authors declare that they have no competing interests.

## Author contributions

P.t.H. and G.C. conceived the study; P.t.H. and R.D.F. drafted the manuscript; all authors contributed further and revised. All authors have approved the final manuscript.

## Supplementary Material

GIGA-D-17-00033_Original_Submission.pdf

GIGA-D-17-00033_Revision_1.pdf

GIGA-D-17-00073_Original_Submission.pdf

Response_to_Reviewer_Comments_Original_Submission.pdf

Reviewer_1_Report_(Original_Submission).pdf

Reviewer_2_Report_(Original_Submission).pdf
