# Supplementary material for: The metagenomic data life-cycle: standards and best practices
Source: Gigascience. 2017 Jun 16;6(8):1–11. doi: 10.1093/gigascience/gix047 (PMC5737865; doi:10.1093/gigascience/gix047)
Supplement: GIGA-D-17-00033_Original_Submission.pdf [file gix047_giga-d-17-00033_original_submission.pdf]

Manuscript
Click here to download Manuscript Metagenomicsdatastandardsenvironment10022017_GigaScie

Title

The metagenomic data life-cycle: standards and best practices

Authors

Petra ten Hoopen[1], Robert D. Finn[1], Lars Ailo Bongo[2], Erwan Corre[3], Bruno Fosso[4], Folker Meyer[5], Alex Mitchell[1], Eric Pelletier[6,7,8], Graziano Pesole[4,9], Monica Santamaria[4], Nils Peder Willassen[2], and Guy Cochrane[1]*

[1]European Molecular Biology Laboratory, European Bioinformatics Institute, Wellcome Genome Campus, Hinxton, Cambridge CB10 1SD, United Kingdom

[2] UiT The Arctic University of Norway, Tromsø N-9037, Norway

[3] CNRS-UPMC, FR 2424, Station Biologique, Roscoff 29680, France

[4] Institute of Biomembranes and Bioenergetics, CNR, Bari 70126, Italy

[5] Argonne National Laboratory, Argone IL60439, USA

[6] Genoscope, CEA, Évry 91000, France

[7] CNRS / UMR-8030, Évry 91000, France

[8] Université Évry val d'Essonne, Évry 91000, France

[9]University of Bari "A. Moro", Bari 70126, Italy

* corresponding author

Guy Cochrane

European Molecular Biology Laboratory

European Bioinformatics Institute

Wellcome Genome Campus

Hinxton

Cambridge CB10 1SD

United Kingdom

Tel: +44(0)1223 494444

Email: cochrane@ebi.ac.uk

Email addresses:

AM: mitchell@ebi.ac.uk

BF: b.fosso@ibbe.cnr.it

EC: erwan.corre@sb-roscoff.fr

EP: eric.pelletier@genoscope.cns.fr

FM: folker@anl.gov

GC: cochrane@ebi.ac.uk

GP: g.pesole@ibbe.cnr.it

LAB: lars.ailo.bongo@uit.no

MS: m.santamaria@ibbe.cnr.it

NPW: nils-peder.willassen@uit.no

PTH: petratenhoopen@yahoo.co.uk

RDF: rdf@ebi.ac.uk

Abstract

Metagenomics data analyses from independent studies can only be compared if the analysis workflows are described in a harmonised way. In this overview, we have mapped the landscape of data standards available for the description of essential steps in metagenomics: (1) material sampling, (2) material sequencing (3) data analysis and (4) data archiving & publishing.

Taking examples from marine research, we summarise essential variables used to describe material sampling processes and sequencing procedures in a metagenomics experiment. These aspects of metagenomics dataset generation have been to some extent addressed by the scientific community but greater awareness and adoption is still needed.

We emphasise the lack of standards relating to reporting how metagenomics datasets are analysed and how the metagenomics data analysis outputs should be archived and published. We propose best practice as a foundation for a community standard to enable reproducibility and better sharing of metagenomics datasets, leading ultimately to greater metagenomics data reuse and repurposing.

Keywords

Metagenomics, metadata, standard, best practice, sampling, sequencing, data analysis.

Background

Recent technological advances allow researchers to examine communities of organisms using such methods as metagenomics, metatranscriptomics and metaproteomics (**Figure 1)**, enabling comprehensive insights into community composition and function. The increased popularity of these meta-omics methods, driven not least by ever decreasing cost, leads to increasing scale and complexity of experimental data and in approaches to their analysis. In addition, there is growing demand for comparisons between communities that have been studied independently, often using very different approaches. However, meaningful interpretation across studies (either through aggregation and interpretation of existing published analyses or through meta-analysis of published experimental data using a uniform method) is challenging. A number of reasons exist for this: (1) each 'omic' analysis workflow is a complex process, consisting of disparate and diverse tasks, ranging from sample collection and processing to data generation and analysis, where each task has many parameters that can affect analysis outputs (for example, it has been shown that a major factor explaining correlations within metagenomics datasets can be DNA preparation and sequencing, [1]); (2) each variable is frequently recorded in a non-standardised way, or not recorded at all; (3) presentation formats of the produced omics data are not unified; (4) omics experimental data and related analysis outputs are either dispersed in several public repositories, or not archived at all.

Here, we review the workflow for metagenomics data generation and analysis. Where possible, we specify essential parameters in the workflow and advise on standardised

systematic reporting of these as variables. We build on the expertise of major public genomic and metagenomic resources: the European Nucleotide Archive (ENA) [2] and EMBL-EBI Metagenomics (EMG) [3] at the EMBL European Bioinformatics Institute in UK; MG-RAST [4] at Argonne National Laboratory in USA; and the extensive knowledge bases in metagenomics available at research centers of excellence, the UiT in Norway, Genoscope in France, SB-Roscoff in France and CNR in Italy.

For the purposes of this paper, we will predominately use marine metagenomics as a 'use case' to highlight the standards environment that we describe. However, we believe that these examples will broadly translate to all areas of metagenomics research, regardless of the environment under study. From the outset, we stress that we do not wish to promote a specific workflow, but rather to demonstrate the importance of having systematic reporting conventions that accurately describe any chosen workflow, from sampling through to the presentation of analysis outputs. Our aim is to describe conventions and standards that are inclusive and extensible, and able to cope with evolving scientific developments in the field. Furthermore, where a given standard has not emerged, we will point to, or propose, a generalised 'best practice' that can be used in its place. While this may produce a foundation from which a new standard could be proposed, any additional formal scientific standards need to come from the community and be ratified by scientific bodies, such as the Genomics Standards Consortium (GSC) [5].

[Figure 1]

Overview of the metagenomics data model

The introduction of new generation sequencing technologies has enabled even small research groups to generate large-scale sequencing data. The resultant DNA sequences and associated information are typically captured in several interconnected objects (**Figure 2**), which represent the following concepts:

- **Study**: Information about the scope of a sequencing effort that groups together all data of the project.
- **Sample**: Information about provenance and characteristics of the sequenced samples.
- **Experiment**: Information about the sequencing experiments, including library and instrument details.
- **Run**: An output of a sequencing experiment containing sequencing reads represented in data files.
- **Analysis**: A set of outputs computed from primary sequencing results, including sequence assemblies, functional and taxonomic annotations.

[Figure 2]

Information associated with DNA sequence is frequently referred to as **'metadata'.** This includes all information described in the study, sample, experiment and run data objects. **Primary data** represent, in this context, primary experimental sequence reads produced by sequencing machines, sometimes with some basic level of quality control (filtering out of poor-quality reads, clipping of low-quality regions, etc.). Following this, for some

metagenomics studies, the **primary data** are analysed directly (e.g. 16S or 18S rRNA gene amplicon studies), while in others, they are assembled into contigs before undergoing further analysis. Regardless of the approach, the output of any computational analysis process (including assembly) on the primary data are here referred to as **derived data**. We discuss derived data in more detail below, but the more harmonised the formats and validations for data and metadata objects, the more easily the generated data be shared, discovered, re-used and re-purposed.

Each metagenomics study has a scope, aim and one or more (human) contributors in each step of the workflow, who may be distributed over a wide geographical area. It is essential to capture contextual information regarding the contributors, since this supports appropriate attribution and credit , and clarifies the responsible parties for each step of the workflow. Contributors to (1) material sampling (2) primary data generation and (3) derived data generation should always be clearly presented in data records. Minimum metadata checklists frequently do not specifically capture data generating or contributing institutions. However, this information is frequently available and can be parsed from the registration systems for reporting individual steps of the data generation workflow or from associated peer-reviewed publications.

Sampling

The method of collecting a sample (a fundamental unit of material isolated from the surrounding environment) is dictated by the nature of the community under investigation,

the environment in which it is found and the type of 'omics' investigation being performed. The slightest deviation in method, regardless of the protocol chosen, can have a profound impact on the final 'omics' analysis results. It is therefore essential that the details of the sampling process are captured accurately and in a standardised way.

Domain experts are in the best position to formulate opinions on the general scope and content of contextual data (environmental characteristics observed or measured during sample collection) and methodological variables (such as sampling volume and filtration method). These opinions are conventionally formalised as data reporting standards by community initiatives such as the GSC for genomics data [5], the Proteomics Standards Initiative (PSI, [6]) for proteomics data or Geo Bon [7] for biodiversity data [8].

The Minimum Information about Metagenomic Sequence (MIMS, [9]) is a GSC-developed data-reporting standard, designed for accurate reporting of contextual information for samples associated with metagenomic sequencing, and is also largely applicable to metatranscriptomics studies. Minimum Information about a MARKer gene Sequence (MIMARKS, [10]) is another GSC-developed contextual data reporting standard for reporting information about a metabarcoding study, which is referred to in the standard as the 'MIMARKS-survey investigation type'.

MIMS and MIMARKS are a part of a broader GSC standard, the Minimum Information of any (x) Sequence (MIxS) [11], which describes 15 different environmental packages that can be used to specify the environmental context of a sequenced microbial community, such as air,

water or host organism-associated. The MIxS descriptors can be combined with any environmental package and together provide rich information on sampling context.

To illustrate, **Table 1** summarises the minimum set of elements required for description of a metagenomic sample taken from an aquatic environment. It uses MIMS mandatory descriptors, combined with the mandatory descriptors of the Water Environment package. Similarly, a sample taken from a gut of a fish host can be described using the MIMS core descriptors, in combination with descriptors in the Host-associated Environment package. The 15 different environmental packages defined by the GSC are available from the GSC website as a single bundled download [12] and are presented in a host of informatics tools that support data reporting and presentation, such as the submission tools of the databases of the International Nucleotide Sequence Database Collaboration [13] and ISAtools [14]. It remains up to the experimentalist to choose the most appropriate package from within the checklist bundle for their study, thereby defining the list of fields that will be used to capture relevant metadata. Before embarking on a metagenomics study, we recommend that the appropriate checklist be identified, so that the appropriate metadata can be captured during the experiment, rather than retrospectively having to determine these metadata.

[Table 1]

The M2B3 data reporting and service standard [15] specifically addresses contextual data relating to marine microbial samples. It represents a common denominator of contextual data from data standards used in the public genomic data archives (MIxS, Version 4.0, [12]),

pan-European network of oceanographic data archives (CDI schema, Version 3.0, [16]) and pan-European network of biodiversity data resources (OBIS schema, Version 1.1, [17]). This M2B3 unified data standard significantly simplifies contextual data reporting, since it provides an interoperable solution for sharing contextual data across data archives from different scientific domains. A minimum M2B3 checklist for reporting contextual data associated with marine microbial samples is summarised in **Table 2**.

[Table 2]

For most adopted standards of this type, only a few contextual data are mandatory, reflecting the balance between usability for the experimentalist reporting his/her science and consumers re-using this science; limiting the number of mandatory fields lowers the burden for experimentalists to comply with the standard, while a small number of parameters are universally, or near-universally, required for downstream analysis. The importance of the optional MIxS and M2B3 fields for metagenomic data analysis is detailed in **Table 3.**

For omics studies it can be useful to introduce a sample attribute, 'biological replicate', where the attribute value is the accession number of the related biological sample. In contrast, 'technical replicates', for which only a single sample exists, are treated downstream in the workflow.

Consistent and rich contextual data can become a powerful tool for metagenomics data analysis. Two marine studies, the *TARA* Oceans sequencing study (PRJEB402, [18]) and

Ocean Sampling Day (OSD, PRJEB5129, [19]) both use the same M2B3 contextual data reporting standard enabling comparison of data within and across studies. For instance, data from the *TARA* Oceans shotgun sequencing of the prokaryotic fraction filtered from seawater (PRJEB1787, [20]) can be compared to the shotgun data from OSD (PRJEB8682, [21]), enabling detailed or complex queries. Specifically, a taxonomic or functional profile from the *TARA* Oceans sample from depth 5m and salinity 38psu (SAMEA2591084, [22]) can be compared to profiles of the OSD sample from the depth 5m (SAMEA3275502, [23]) or the OSD sample with the same salinity 38psu (SAMEA3275531, [24]). In contrast, very few conclusions can be drawn from a comparison to a sample with insufficient contextual information (SAMN00194025, [25]).

[Table 3]

Details of the project investigators are usually recorded in the Study metadata object and sampling contextual data are mostly captured in the Sample metadata object, **Figure 2**. A common way to standardise reporting of contextual data is via a checklist of key-value pairs, thereby ensuring parameters of a similar kind are described consistently. Furthermore, syntactic and semantic rules can be pre-defined in the checklist, enabling validation of compliance with these rules. For instance, automated checks can be applied to test whether a mandatory descriptor (key) in the checklist has a value and whether the value is in a specified format. Each element to be checked can be pre-defined as text, a class or term from an ontology, a controlled vocabulary or taxonomic index, or formulated as a regular expression. (Regular expressions can be used, for example, to check that the key ‘collection

date and time’ complies with ISO 8601 standards and that numeric values lie within a defined range.)

The most common formats for sharing Study and Sample metadata are XML, TSV, ISA-tab or JSON formats. Examples of the Study and Sample XML are available from the European Nucleotide Archive [26], [27], where the files are also validated against the XML schema [28]. Regardless of the format used to supply the metadata, because they all use the same underlying standards, a simple translation between the formats enables different data to be compared. This allows scientists to use different tools or approaches that they are most familiar with, whilst ensuring consistent delivery of the metadata.

Sequencing

Once a sample is collected and its provenance recorded, it is subjected to preparation steps for nucleotide sequence analysis. This may happen immediately after sampling, or in stages over many months. Processing steps cover all handling of the sample leading to the DNA isolation. Although MIxS covers some of the metadata fields for reporting the DNA extraction steps, it is extremely difficult to define a generic set of fields describing the DNA extraction method with a high granularity due to its complexity and diversity. For example, it might be relatively straightforward to identify variables for reporting isolation of DNA from a seawater sample but that will not suit the more complex DNA isolation procedure for a sediment sample. We suggest the best practice here is to use the existing MIxS fields, such as the *sample material processing*, *nucleic acid extraction* and *nucleic acid amplification* for

concise description of the nucleic acid preparation. A detailed description, or a reference to the material preparation steps, is important due to the significant influence this can have on the observed profile of the microbial community under investigation.

Equally critical for the downstream metagenomic data analysis and interpretation is the reporting of sequencing library preparation protocols and parameters as well as sequencing machine configurations.

Table 4 shows mandatory descriptors for new generation nucleotide sequencing experiments as currently captured by INSDC databases. **Table 5** lists non-mandatory descriptors including MIMS sequence-related descriptors and provides our opinion on the importance of these descriptors for metagenomic data analysis. Note that while a number of controlled vocabularies have been developed for accurate recording of sequencing experiment parameters, the evolution of these constrained vocabularies is very dynamic and driven by technological advances.

[Table 4]

[Table 5]

Variable parameters of the library preparation and instrumentation are captured in the metadata objects Experiment and Run (see **Figure 2**). Examples of the Experiment and Run XML are available, for example, from the ENA [29],[30]. Each Experiment should refer to

Study and Sample objects, to provide context for the sequencing, and is referred to from the Run objects, which point to the primary sequencing reads.

The **primary data** (the reads) are stored in files of various formats, which can be standard (BAM, CRAM or Fastq) or a platform-specific, as with SFF, PacBio, Oxford Nanopore or Complete Genomics. Information on the read data format must be indicated in the description of sequencing.

The minimum information encapsulated in read data files are base calls with quality scores. Quality requirements on read data files are file format-specific and are summarised, for example, in the ENA data submission documentation [31]. A freely available diagnostic tool for validation of CRAM and BAM files is the Picard ValidateSamFile [32]. Validation of Fastq files is less straightforward since there is no single FASTQ specification. Recommended usage of FASTQ can be found for instance in the ENA guidelines [33]. An open resource for managing next generation sequencing datasets is the NGSUtils [34], which also contains tools for operations with FASTQ files. As sequencing technologies change over time the formats and associated validation tools may well change, so a comprehensive list of formats and tools is likely to become outdated. The key point is to adopt a widely used format and to check for file format and integrity (e.g. checksums).

Analysis

Standards in metagenomics for the description of sampling and sequencing have grown out of those from more traditional genomics. While there are still some shortcomings in these standards, as highlighted in the previous sections, metadata concerning sampling and sequencing are commonly captured for metagenomics studies. Compliance is high partly due to the scientific journals requiring scientist to submit sequence data to an INSDC database prior to publication. However, there are currently no standards for reporting how metagenomics datasets have been analysed. While systematic analysis workflows, such as those offered by EMG, IMG/M [35] META-pipe [36] and MG-RAST, provide a standard that is documented (albeit in different ways), many published datasets are analysed by in-house bespoke pipelines. Although many authors provide an outline in the 'materials and methods' or 'supplementary materials' section of their publications, it is rarely possible to reproduce the analysis from this alone, due to missing software parameters, lack of detail on software versions and ambiguous reference databases and their associated versions.

Typically, once the sequence read files have been produced, they are analysed using one or more workflows [37], with each workflow comprising different data processing or analysis components. Most workflows involve aspects such as quality control (for example, removing sequences that fail to meet predefined quality scores), assembly, sequence binning (e.g. identifying 16S rRNA genes or protein coding sequences), and taxonomic classification of sequences and/or functional prediction. However, each workflow will be tailored to how the sample has been processed and the question being addressed. For example, if a sample has been size-fractioned for viruses, using a 0.22 µm filter, there would be little point analysing

the data for eukaryotic 18S rRNA, as any eukaryotic organisms would have been physically removed from the sample before the DNA extraction process.

Analysis workflows typically have one or more of the following components:

(i) Central algorithmic software, which may be from a third party source.

(ii) 'Glue' software that may ensure input/output formats, or split/join input files for parallelisation.

(iii) Reference datasets that are used by (i). For example, the Greengenes database of 16S rRNA genes [38], SILVA database of 16S/ 18S SSU ribosomal RNA genes [39] and the NCBI non-redundant database of non-identical protein sequences [40], for taxonomic or functional analysis.

However, even knowing these elements may not be sufficient for analyses to be independently re-created. For example, the algorithm may accept a set of input parameters that can be used to fine-tune an analysis, such as selecting an E-value threshold for determining significance of a sequence match to a reference database. Other parameters may influence speed-performance, which allows the original analysis to complete in a timely fashion, but they may or may not have an effect on the results. For example, running *hmmsearch* from the HMMER package, changing the number of CPUs used will not change the results, but changing options on the heuristics such at the --F1 threshold (which controls the number of sequences passing the first heuristic state) may alter the output; both will potentially increase performance in terms of speed.

Capturing and reporting *all* provenance information is essential to understand exactly what analysis has been performed on the data, and to ensure reproducibility [41]. The use of publicly available analysis pipelines (such as EMG, MG-RAST or META-pipe) helps with this process, since analysis is performed using pre-defined components, settings and databases (or, in some cases, using user-selected components, selected from a predefined list of options). Nevertheless, capturing analysis metadata remains essential as, for example, MG-RAST allows the users to dynamically set E-value thresholds after the pipeline analysis has been performed. Furthermore, the tools, libraries, and reference databases used by the pipelines are regularly updated, and thus capturing analysis provenance information is vitally important and should be systematically 'tagged' to the results.

To date, there is no universally endorsed 'Analysis standard' for describing and recreating a metagenomics analysis pipeline, and without this standard (and subsequent adoption/enforcement) it will continue to be difficult, if not impossible, to reproduce analysis workflows. However, all is not lost. 'Workflows' and their definitions is an active field of computer science research, and potential solutions are already available, including Common Workflow Language (CWL), Yet Another Workflow Language (YAWL), Business Process Execution Language (BPEL) and Microsoft Azure's Workflow Definition Language to name but a few. Several of the co-authors for this publication already participate in the GSC M5 consultation group, which aims to define a standard enabling the recreation and exchange of metagenomics data sets. In the absence of a standard, we believe it is important to define some of the basic best practices, from which an accepted standard would formally encapsulate. For simplicity, we will focus on a single 'best practice' use case:

the description of the analysis of a run. Other types of analysis, such as pooling of runs or comparing results between runs are beyond the scope of this article.

A schematic overview of a best practice for analysis metadata collection is shown in **Figure 3A**. An overarching set of metadata relating to analysis should encapsulate generic information such as analysis centre, name of bioinformaticians, analysis objectives, name of overall analysis (if appropriate) and the date on which the analysis was performed. It should also contain appropriate pointers to the run data, run sequence metadata and associated sample data. Underneath the overarching analysis metadata is a collection of analysis components, which describe each stage of the analysis **Figure 3B**. Each component can be divided into three sections: input(s), analysis algorithm and output(s).

The input section should describe the details of the various inputs to the analysis, which could be the raw sequence reads or the output of another analysis component, reference databases and their provenance data, such as version, where necessary. The analysis section should contain the algorithm tool, version, all parameters used and a basic description of the analysis. The output section should describe each output from the analysis, together with a description of contents and format.

Each analysis component could then be coupled to form an analysis workflow as shown in **Figure 3C**. The workflow may be in a portable intermediate format that can be submitted to a workflow manager for execution in a specific environment.

[Figure 3]

This best practice framework is merely that - a best practice, and we have not touched on the technical issues of how to capture this information or on controlled vocabularies (since these need to come from the community). Furthermore, enforcing compliance and validation against the standard will also require a community effort. Complete validation would require the standard to be machine readable and deployable, with potentially the need to have small 'test' datasets and their associated results, to perform regression testing of the analysis metadata. However, who is responsible for validation and what happens if something fails after publication are open questions. This could arguably be a step too far; currently sampling and sequence metadata are validated against the standard, but taken in good faith to be correct beyond this.

Analysis Results Archiving - a final piece?

Having an analysis provenance standard would allow metagenomics analysis results to be recreated more readily. While this is undoubtedly an important and necessary step, it has a major limitation within the community. As indicated [42], the fraction of money spent on informatics from an overall project budget is increasing dramatically. Metagenomics datasets tend to be large, in the order of GB-TB and processing may take 1000s of CPU hours, restricting reanalysis to only those with significant compute resources. For example, the subset of the *TARA* Oceans Ocean Microbiome Project (PRJEB7988, [43]) that has been size-fractioned for prokaryotes comprises 135 samples with 248 runs containing 28.8 billion reads. The analysis output represents about 10TB of data with 23.2 billion predicted protein

coding sequences. Thus, reanalysis would be costly and potentially wasteful if a particular workflow had already been run on the data. Therefore, a final step in a metagenomics analysis is the appropriate archiving of results. There is an obvious cost-benefit balance to be drawn here, as storing every intermediate of a workflow would lead to an explosion of data. Clearly, key intermediates and outputs of an analysis workflow need to be determined. These key archived components will be tailored to the analysis, but should at least include operational taxonomic unit (OTU) counts and assignments, functional assignment counts and read/sequence positional information for the aforementioned assignments. Such data files are already made available from MG-RAST and EMG and those from other sources are accepted for archiving within ENA.

If metagenomic assemblies have been performed, then these should have an appropriate structure of contigs, scaffolds or chromosomes with an appropriate format as detailed for example in the ENA data submission documentation [44]. Due to the overheads of producing an assembly, these should be archived, ideally with an INSDC database.

The data model for metagenomics, as described in **Figure 2**, represents metagenomic analysis results in the data Analysis object with appropriate pointers to the corresponding run sequence metadata and associated sample collection contextual data. While there is an established practice to archive primary sequence data in the Run object and assemblies of the primary sequences in the Analysis object, it is not a common practice to archive results of functional and taxonomic metagenomic analysis of in-house bespoke pipelines. It would be beneficial to the metagenomics community to include this into the best practice and such data are accepted by ENA for archiving. The metagenomics standard environment reviewed

here as well as outcomes of the GSC M5 consultation group can contribute to defining required descriptors of the Analysis object for archiving of metagenomics analysis results, which can serve as a framework for exchange of metagenomics data sets on a routine bases, similarly as is currently done for the primary sequence data.

Future

One challenge over the next several years will be the validation of compliance across the entirety of the standards and best practise that we have covered. While validation tools and recommended practises exist for parts (e.g. contextual data descriptors using MIxS-compliant validation tools from ISA and experimental descriptors upon submission to an INSDC database), not all parts have such maturity (e.g. analysis descriptors) and there exists no overarching validation protocol for an entire metagenomics study. The GSC is aiming to contribute in this area with the introduction of MIxS “profiles”, to provide an overlay on top of MIxS environmental packages and the core MIxS fields. These profiles will enable the creation of tool suites for compliance checking. In addition (and perhaps more importantly) they will enable groups of researchers, institutes, funders and other communities to define levels of compliance for contextual data sets. Examples of this are the NSF NEON [45] and the NSF CZO [46] networks that are working with the GSC to establish silver, gold and platinum sets of parameters that need to be provided and validated for data sets to be compliant. A key moment in the acceptance of said new profiles will be the availability of tools support for data creators, end-users and portals. Imagine, for instance, a search for data sets in EMG or MG-RAST that allows restriction of the search to just platinum-level data

sets. For the data consumer this will result in better ways of telling their science story using third party data and for the data creator this will provide guidance on what to create for a specific community. In addition funding agencies can require certain minimal compliance.

A further area to be addressed is that of standards around descriptions of metagenomics analyses. The creation of a lightweight data standard for an analysis object that allows easy transfer of analyses is a key goal of the GSC M5 initiative but the complexity of the task and lack of dedicated resourcing has rendered progress slow; while frameworks and systems for recording analysis provenance need to be established, we have aimed to indicate in this publication a set of best practice that can form the foundation for a community standard enabling the recreation and exchange of metagenomics data sets. Improving standardisation will also help raise clarity in the literature around metagenomics through a tightening of language. For example, UProC [47] uses Pfam [48] matches as a reference library with the results being referred to as a 'Pfam hit'. However, this may not necessarily be a Pfam hit, as a Pfam hit is defined as a sequence match scoring greater than Pfam defined threshold to the Pfam profile Hidden Markov Model (i.e. the Pfam database method).

Conclusions

In this overview of the metagenomics standard environment we have outlined best practice for the reporting of metagenomics workflows. We have reviewed the essential steps: (1)

material sampling, (2) material sequencing (3) data analysis and (4) data archiving, and highlighted essential variable parameters and common data formats in each step.

Reporting on the provenance of a sample and associated nucleotide sequence data is largely established by public sequence data repositories and is also being addressed by contextual data standardisation initiatives. In contrast, a reporting standard on metagenomics data analysis is absent, yet the high complexity of metagenomics creates a pressing demand for establishing such a practice. Capturing key metadata relating to analysis would greatly improve reproducibility. Archiving key results of the metagenomics data analysis would allow a more accurate evaluation of the benefits of reproducing the analysis.

Only by adopting these standards and best practices can metagenomics data be assessed agains the FAIR (Findable, Accessible, Interoperable and Reusable) principles that should be applied to any scientific dataset [49].

List of abbreviations

BAM: Binary Alignment/Map

BPEL: Business Process Execution Language

CDI: Common Data Index

CHEBI: Chemical Entities of Biological Interest

CNR: Consiglio Nazionale delle Ricerche

CPU: Central Processing Unit

CRAM: Compression Reduced Alignment/Map

CWL: Common Workflow Language

EBI: European Bioinformatics Institute

EMBL: European Molecular Biology Laboratory

EMG: EBI Metagenomics

ENA : European Nucleotide Archive

ENVO: Environment Ontology

GEO BON: Group on Earth Observations Biodiversity Observation Network

GO: Gene Ontology

GSC: Genomic Standards Consortium

GSC M5: Genomic Standards Consortium M5 working group

IMG/M: Integrated Microbial Genomes with Microbiomes

INSDC: International Nucleotide Sequence Database Collaboration

ISO: International Standards Organisation

JSON: JavaScript Object Notation

MG-RAST: Metagenomic Rapid Annotations using Subsystems Technology

MIMS: Minimum Information about a Metagenome Sequence

MIMARKS: Minimum Information about a MARKer gene Sequence

MIxS: Minimum Information about any (x) Sequence

M2B3: Marine Microbial Biodiversity Bioinformatics and Biotechnology

NCBI: National Center for Biotechnology Information

NSF NEON: National Science Foundation National Ecological Observatory Network

NSF CZO: National Science Foundation Critical Zone Observatory

OBIS: Ocean Biogeographic Information System

OSD: Ocean Sampling Day

OTU: Operational Taxonomic Unit

PSI: Proteomics Standards Initiative

SDN: SeaDataNet

SFF: Standard Flowgram Format

SSU: Small Subunit ribosomal RNA

TB: Terabyte

TSV: Tab Separated Values

UiT: Universitetet i Tromsø

UProC: Ultrafast Protein Classification

UTC: Coordinated Universal Time

XML: Extensible Markup Language

YAWL: Yet Another Workflow Language

Declarations

Ethics approval and consent to participate

Not applicable

Consent for publication

Not applicable

Competing interests

The authors declare that they have no competing interests.

Funding

This work was supported by the ELIXIR-EXCELERATE funded by the European Commission within the Research Infrastructures programme of Horizon 2020, grant agreement number 676559.

Author's contribution

PtH and GC conceived the study; PtH and RDF drafted the manuscript; all authors contributed further and revised. All authors have approved the final manuscript.

References

[1] Salter SJ, Cox MJ, Turek EM, Calus ST, Cookson WO, Moffatt MF, et al. Reagent and laboratory contamination can critically impact sequence-based microbiome analysis. BMC Biology. 2014;12:87.

[2] Toribio AL, Alako B, Amid C, Cerdeño-Tárraga A, Clarke L, Cleland I, et al. European Nucleotide Archive in 2016. Nucleic Acids Res. 2016; doi:10.1093/nar/gkw1106.

[3] Mitchell A, Bucchini F, Cochrane G, Denise H, Hoopen PT, Fraser M, et al. EBI metagenomics in 2016 - an expanding and evolving resource for the analysis and archiving of metagenomic data. Nucleic Acid Res. 2016;44:D595-D603.

[4] Meyer F, Paarmann D, D'Souza M, Olson R , Glass EM, Kubal M, et al. The Metagenomics RAST server – a public resource for the automatic phylogenetic and functional analysis of metagenomes. BMC Bioinformatics. 2008;9:386.

[5] Field D, Amaral-Zettler L, Cochrane G, Cole JR, Dawyndt P, Garrity GM et al. The Genomic Standards Consortium. PLoS Biol. 2011;9(6):e1001088.

[6] Orchard S., Hermjakob H. and Apweiler R. (2003) The Proteomics Standards Initiative. Proteomics. 2003;3(7):1374-6.

[7] The Group on Earth Observations Biodiversity Observation Network. http://geobon.org/essential-biodiversity-variables/connect-with-geoss/. Accessed 19 Jan 2017.

[8] Pereira HM, Ferrier S, Walters M, Geller GN, Jongman RHG, Scholes RJ et al. Essentail Biodiversity Variables. Science. 2013;339(6117):277-8.

[9] The Minimum Information about a Metagenome Sequence. http://wiki.gensc.org/index.php?title=MIGS/MIMS. Accessed 19 Jan 2017.

[10] The Minimum Information about a Marker Gene Sequence. http://wiki.gensc.org/index.php?title=MIMARKS. Accessed 19 Jan 2017.

[11] Yilmaz, P, Gilbert, JA, Knight, R, Amaral-Zettler L, Karsch-Mizrachi I, Cochrane G et al. The Genomic Standards Consortium: bringing standards to life for microbial ecology. The ISME J. 2011;**5**,1565-7.

[12] The Minimum Information about any (x) Sequence, Version 4.0. http:// wiki. gensc. org/ index. php? title= MIxS. Accessed 19 Jan 2017.

[13] Cochrane G, Karsch-Mizrachi I, Takagi T and International Nucleotide Sequence Database Collaboration. The International Nucleotide Sequence Database Collaboration. Nucleic Acid Res. 2016;44:D40-D50.

[14] The ISA framework and tools. http://isa-tools.org/. Accessed 7 February 2017.

[15] Ten Hoopen P, Pesant S, Kottman R, Kopf A, Bicak M, Claus S et al. Marine microbial biodiversity, bioinformatics and biotechnology (M2B3) data reporting and service standards. The Standards in Genomic Sciences 2015;10:20.

[16] The Common Data Index, Version 3.0. http:// www. seadatanet. org/ Data-Access/ Common-Data-Index-CDI. Accessed 19 Jan 2017.

[17] The Ocean Biogeographic Information System data standard, Version 1.1. http://old.iobis.org/node/304. Accessed 19 Jan 2017.

[18] The *TARA* Oceans umbrella project record of barcoding and shotgun sequencing. http://www.ebi.ac.uk/ena/data/view/PRJEB402. Accessed 7 Feb 2017.

[19] The Ocean Sampling Day umbrella project record of amplicon and metagenome sequencing. http://www.ebi.ac.uk/ena/data/view/PRJEB5129. Accessed 7 Feb 2017.

[20] The record of the *TARA* Oceans shotgun sequencing project of the prokaryotic fraction filtered from seawater. http://www.ebi.ac.uk/ena/data/view/PRJEB1787. Accessed 7 Feb 2017.

[21] The record of the Ocean Sampling Day shotgun sequencing project from the year 2014. http://www.ebi.ac.uk/ena/data/view/PRJEB8682. Accessed 7 Feb 2017.

[22] The record of a *TARA* Oceans sample from depth 5m and salinity 38psu. https://www.ebi.ac.uk/metagenomics/projects/ERP001736/samples/ERS477979. Accessed 7 Feb 2017.

[23] The record of an Ocean Sampling Day sample from depth 5m. https://www.ebi.ac.uk/metagenomics/projects/ERP009703/samples/ERS667511. Accessed 7 Feb 2017.

[24] The record of an Ocean Sampling Day sample with salinity 38psu. https://www.ebi.ac.uk/metagenomics/projects/ERP009703/samples/ERS667548. Accessed 7 Feb 2017.

[25] The record of an oil spill water sample from Gulfport. http://www.ebi.ac.uk/ena/data/view/SAMN00194025. Accessed 7 Feb 2017.

[26] An example of a study XML. http://www.ebi.ac.uk/ena/submit/preparing-xmls#study. Accessed 19 Jan 2017.

[27] An example of a sample XML. http://www.ebi.ac.uk/ena/submit/preparing-xmls#sample. Accessed 19 Jan 2017.

[28] The validating XMLs document. http://www.ebi.ac.uk/ena/submit/validating-xmls. Accessed 19 Jan 2017.

[29] An example of an experiment XML. http://www.ebi.ac.uk/ena/submit/preparing-xmls#experiment. Accessed 19 Jan 2017.

[30] An example of a run XML. http://www.ebi.ac.uk/ena/submit/preparing-xmls#run. Accessed 19 Jan 2017.

[31] The document on the ENA-supported read file formats. http://www.ebi.ac.uk/ena/submit/read-file-formats. Accessed 19 Jan 2017.

[32] The document on the Picard set of command line tools. http://broadinstitute.github.io/picard/. Accessed 19 Jan 2017.

[33] The document on recommended usage of FASTQ files. http://www.ebi.ac.uk/ena/submit/read-data-format. Accessed 7 February 2017.

[34] The document on the NGSUtils tools for next-generation sequencing analysis. http://ngsutils.org/. Accessed 19 Jan 2017.

[35] Markowitz VM, Chen IM, Chu K, Szeto E, Palaniappan K, Pillay M, Ratner A et al. IMG/M 4 version of the integrated metagenome comparative analysis system. Nucleic Acid Res. 2015;42:D568-73.

[36] Robertsen EM, Kahlke T, Raknes IA, Pedersen E, Semb EK, Ernstsen M et al. META-pipe – pipeline annotation, analysis and visualisation of marine metagenomic sequence data. https://arxiv.org/abs/1604.04103v1. Accessed 19 Jan 2017.

[37] Leipzig J. A review of bioinformatic pipeline frameworks. Briefings in Bioinformatics. 2016; doi:10.1093/bib/bbw020.

[38] The Greengenes Database. http://greengenes.secondgenome.com/. Accessed 19 Jan 2017.

[39] The Silva Database. https://www.arb-silva.de/. Accessed 19 Jan 2017.

[40] The NCBI nr Database. https://www.ncbi.nlm.nih.gov/refseq/. Accessed 19 Jan 2017.

[41] Huntemann M, Ivanova NN, Mavromatis K, Tripp HJ, Paez-Espino D, Tennessen K et al. The standard operating procedure of the DOE-JGI Metagenome Annotation Pipeline (MAPv v4.). Standards in Genomic Sciences. 2016; doi:10.1186/s40793-016-0138-x.

[42] Sboner A, Mu XJ, Greenbaum D, Auerbach RK and Gerstein MB. The real costs of sequencing: higher than you think! Genome Biology. 2011;12:125.

[43] The record of the *TARA* Oceans Ocean Microbiome Project. http://www.ebi.ac.uk/ena/data/view/PRJEB7988. Accessed 7 Feb 2017.

[44] The document on a genome assembly submission to the ENA. http://www.ebi.ac.uk/ena/submit/genomes-sequence-submission. Accessed 19 Jan 2017.

[45] The National Science Foundation National Ecological Observatory Network. http://www.neonscience.org/. Accessed 19 Jan 2017.

[46] The National Science Foundation Critical Zone Observatory. http://criticalzone.org/national/. Accessed 19 Jan 2017.

[47] Meinicke P. UProC: tools for ultra-fast protein domain classification. Bioinformatics. 2014;31(9):1382-8.

[48] Finn RD, Coggill P, Eberhardt RY, Eddy SR, Mistry J, Mitchell AL et al. The Pfam protein families database: towards a more sustainable future. Nucleic Acid Res. 2016;44:D279-85.

[49] Wilkinson MD, Dumontier M, Aalbersberg IJJ, Appleton G, Axton M, Baak A et al. The FAIR Guiding Principles for scientific data management and stewardship. Sci. Data 2016; doi:10.1038/sdata.2016.18 (2016).

[50] The controlled vocabulary for the INSDC country qualifier. http://www.insdc.org/country.html. Accessed 19 Jan 2017.

[51] The Environment Ontology browser. http://www.environmentontology.org/Browse-EnvO. Accessed 19 Jan 2017.

[52] The SeaDataNet L06 controlled vocabulary of platform categories. http://seadatanet.maris2.nl/v_bodc_vocab_v2/search.asp?lib=L06. Accessed 19 Jan 2017.

[53] The SeaDataNet P02 controlled vocabulary of parameters. http://seadatanet.maris2.nl/v_bodc_vocab_v2/search.asp?lib=P02. Accessed 19 Jan 2017.

[54] The controlled vocabulary of BODC data storage units. http://seadatanet.maris2.nl/v_bodc_vocab_v2/search.asp?lib=P06. Accessed 19 Jan 2017.

[55] The CHEBI ontological classification of small chemical compounds. http://www.ebi.ac.uk/chebi/init.do. Accessed 19 Jan 2017.

[56] The National Center for Biotechnology Information taxonomy index. https://www.ncbi.nlm.nih.gov/taxonomy. Accessed 19 Jan 2017.

[57] The controlled vocabulary for sequencing instrument models. http://www.ebi.ac.uk/ena/submit/preparing-xmls#experiment. Accessed 19 Jan 2017.

[58] The controlled vocabulary for the library source. http://www.ebi.ac.uk/ena/submit/preparing-xmls#experiment. Accessed 19 Jan 2017.

[59] The controlled vocabulary for the library strategy. http://www.ebi.ac.uk/ena/submit/preparing-xmls#experiment. Accessed 19 Jan 2017.

[60] The controlled vocabulary for the library selection. http://www.ebi.ac.uk/ena/submit/preparing-xmls#experiment. Accessed 19 Jan 2017.

Figure Legends

Figure 1: A generalised metagenomics data analysis workflow in the context of other ‘omics’ approaches.

Figure 2: A common data model for read data and associated metadata.

Figure 3: Schematic overview of best practice for analysis metadata collection with example fields.

Tables

Table 1: Checklist of MIMS mandatory descriptors for a sample taken from an aquatic environment and associated with a metagenomic sequencing experiment.

MIMS-mandatory water sample provenance descriptors	descriptor format
submitted to INSDC	boolean
project name	text
investigation type	fixed value: 'metagenome'
geographic location (latitude and longitude)	decimal degrees in WGS84 system
depth	metres: positive below the sea surface
geographic location (country and/or sea region)	INSDC country list [50]
collection date	ISO8601 date and time
environment (biome)	ENVO class [51]
environment (feature)	ENVO class
environment (material)	ENVO class
environment package	MIxS controlled vocabulary [12]

Table 2: Checklist of M2B3 mandatory descriptors for a microbial sample taken from a saline water environment and associated with a metagenomic sequencing experiment.

M2B3-mandatory saline water sample provenance descriptors	descriptor format
INVESTIGATION_campaign	text
INVESTIGATION_site	text
INVESTIGATION_platform	SDN:L06 controlled vocabulary [52]
EVENT_latitude	decimal degrees in WGS84 system
EVENT_longitude	decimal degrees in WGS84 system
EVENT_date/time	ISO8601 date and time in UTC
SAMPLE_title	text
SAMPLE_protocol label	text
SAMPLE_depth	metres; positive below the sea surface
ENVIRONMENT_environment (biome)	ENVO class
ENVIRONMENT_environment (feature)	ENVO class
ENVIRONMENT_environment (material)	ENVO class
ENVIRONMENT_temperature	SDN:P02 [53], SDN:P06 [54] controlled vocab.
ENVIRONMENT_salinity	SDN:P02, SDN:P06 controlled vocab.

Table 3: Selection of non-mandatory MIxS and M2B3 descriptors (column B) and formats (column D). These descriptors cover such areas as the structure or viability of the community under investigation and sample pooling procedures. Column A groups descriptors that are related conceptually (1 – sample collection method & device, 2 – sample processing, 3 – sample quantity, 4 – storage container, 5 – storage duration, 6 – storage temperature, 7 – chemical treatment, 8 – microbial fraction thresholds, 9 – sample content, 10 – pigment concentration, 11 – fluorescence, 12 – density, 13 – organism abundance, 14 – primary production, 15 – bacterial production, 16 – organism biomass, 17 – organism biovolume, 18 – organism size, 19 – investigation contributors, 20 – unique taxonomic index identifier for organism host). Column C shows the descriptor association with the respective contextual data reporting standard suitable for marine metagenomic data. Column E suggests the descriptor's importance for metagenomic data analysis (H – high relevance, M – medium relevance, L – low relevance).

A<br>group	B<br>non-mandatory sample provenance descriptors	C<br>standard	D<br>descriptor<br>format	E<br>value for analysis (H/M/L)
1	sample collection device or method	MIxS(MIMS)	text	H
1	EVENT_device	M2B3	text	H
1	EVENT_method	M2B3	text	H
2	sample material processing	MIxS(MIMS)	text	H
3	amount or size of sample collected	MIxS(MIMS)	numeric & unit	H
3	SAMPLE_quantity (e.g. length, mass)	M2B3	text	H

4	sample storage location	MIxS(water)	text	L
4	SAMPLE_container (e.g. storage container)	M2B3	text	L
5	sample storage duration	MIxS(water)	interval	H
6	sample storage temperature	MIxS(water)	numeric & unit	H
6	SAMPLE_treatment_storage (e.g. temperature)	M2B3	text	H
7	chemical administration	MIxS(water)	CHEBI ontology [55]	M
7	SAMPLE_treatment_chemicals	M2B3	CHEBI ontology	M
8	SAMPLE_size_fraction_upper_threshold	M2B3	text	H
8	SAMPLE_size_fraction_lower_threshold	M2B3	text	H
9	SAMPLE_content (e.g. 0.22 µm filter, 20mL water)	M2B3	text	H
10	concentration of chlorophyll	MIxS(water)	numeric & unit	HM
10	ENVIRONMENT_ecosystem_pigment concentration	M2B3	SDN:P02, SDN:P06 controlled vocab.	HM
11	Fluorescence	MIxS(water)	numeric & unit	HM
11	ENVIRONMENT_ecosystem_fluorescence	M2B3	SDN:P02, SDN:P06 controlled vocab.	HM
12	density	MIxS(water)	numeric & unit	M
13	organism count	MIxS(water)	numeric & unit	ML
13	ENVIRONMENT_ecosystem_picoplankton (flow cytometry) abundance	M2B3	SDN:P02, SDN:P06 controlled vocab.	ML

13	ENVIRONMENT_ecosystem_nano/microplankton abundance	M2B3	SDN:P02, SDN:P06 controlled vocab.	ML
13	ENVIRONMENT_ecosystem_meso/macroplankton abundance	M2B3	SDN:P02, SDN:P06 controlled vocab.	ML
14	primary production	MIxS(water)	numeric & unit	M
14	ENVIRONMENT_ecosystem_primary production	M2B3	SDN:P02, SDN:P06 controlled vocab.	M
15	bacterial production	MIxS(water)	numeric & unit	M
15	ENVIRONMENT_ecosystem_bacterial production	M2B3	SDN:P02, SDN:P06 controlled vocab.	M
16	biomass	MIxS(water)	numeric & unit	ML
16	ORGANISM_biomass	M2B3	numeric & unit & method	ML
17	ORGANISM_biovolume	M2B3	numeric & unit & method	L
18	ORGANISM_size	M2B3	numeric & unit & method	L
19	INVESTIGATION_authors	M2B3	text	M
20	host taxid	MIxS (host associated)	NCBI Taxonomy identifier [56]	M

Table 4: Mandatory descriptors for sequencing.

mandatory descriptors of sequencing provenance	descriptor format
instrument platform	controlled vocabulary [illumina, oxford nanopore, pacbio smrt, ion torrent, ls454, complete genomics, capillary]
instrument model	controlled vocabulary [57]
library source	controlled vocabulary [58]
library strategy	controlled vocabulary [59]
library selection	controlled vocabulary [60]
library layout	controlled vocabulary [single, paired]
read file name	text
read file md5 checksum	32-digit hexadecimal number
second read file name (for paired Fastq files)	text
Second read file md5 checksum (for paired Fastq files)	32-digit hexadecimal number

Table 5: Non-mandatory sequencing descriptors (column A) and formats (column B). Column C suggests the descriptor's potential importance for metagenomic data analysis (H – high relevance, M – medium relevance, L – low relevance).

**A**<br>non-mandatory descriptors of sequencing provenance	**B**<br>descriptor format	**C**<br>value for analysis (H/M/L)
sequencing centre contact	text	M
sequencing experiment name	text	L
library name	text	L
library description	text	L
library construction protocol	text	M
library construction method (MIMS)	text	M
library size (MIMS)	numeric	M
library reads sequenced (MIMS)	numeric	M
library vector (MIMS)	text	M
library screening strategy (MIMS)	text	M
insert size (for paired read files)	numeric	M
spot layout (for SFF read files)	controlled vocabulary (single, paired FF, paired FR)	M
linker sequence (for SFF read files)	sequence of nucleotides	H
multiplex identifiers (MIMS)	sequence of nucleotides	H

adapters (MIMS)	sequence of nucleotides	H
quality scoring system (for Fastq files)	controlled vocabulary (phred, log-odds)	H
quality encoding (for Fastq files)	controlled vocabulary (ascii, decimal, hexadecimal)	H
ascii offset (for Fastq files)	controlled vocabulary (!, @)	H
nucleic acid extraction SOP (MIMS)	text	H
nucleic acid amplification SOP (MIMS)	text	H
sequencing coverage	numeric	H

Click here to download Figure Figure1.pdf

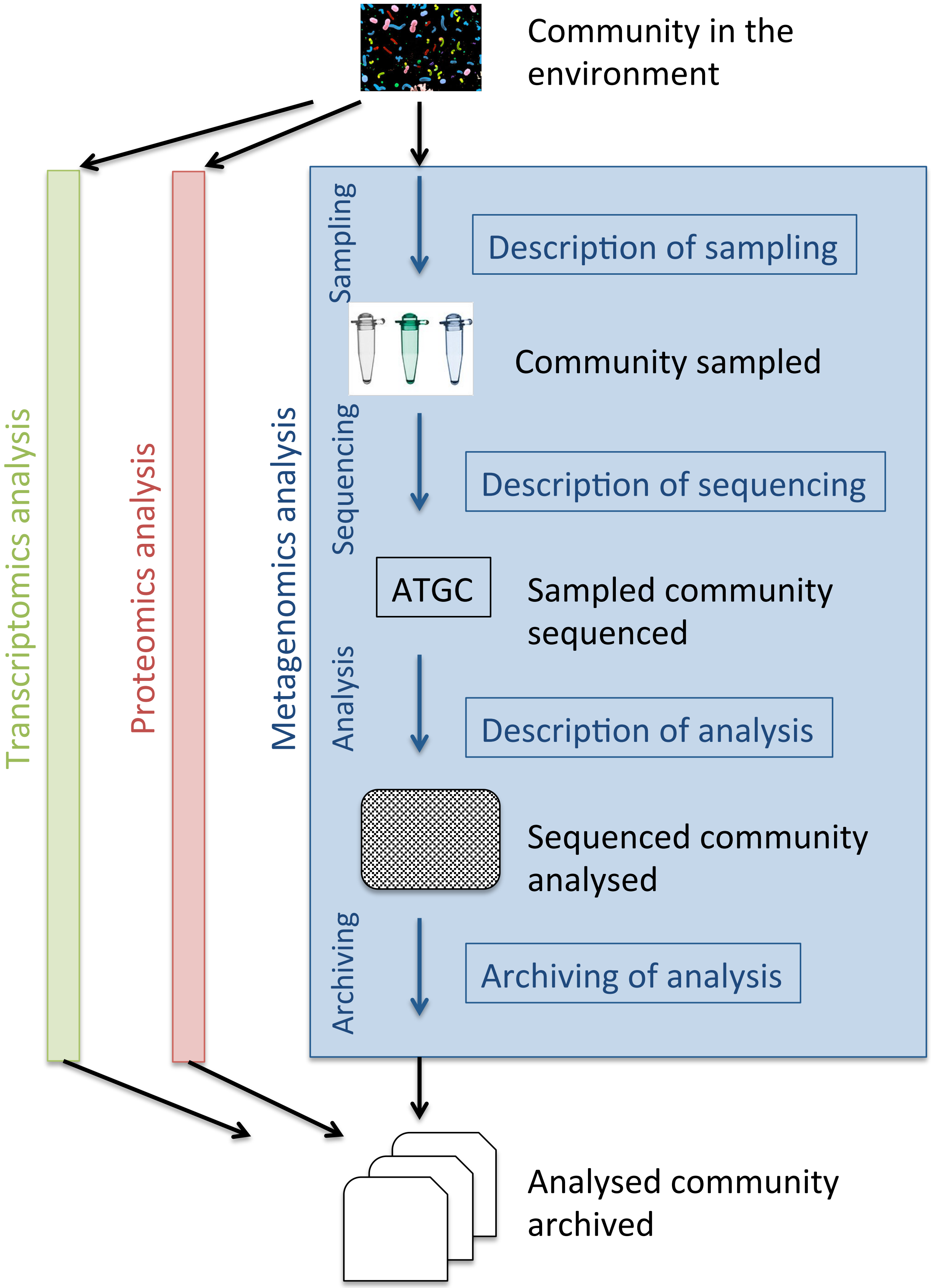

Click here to download Figure Figure2.pdf

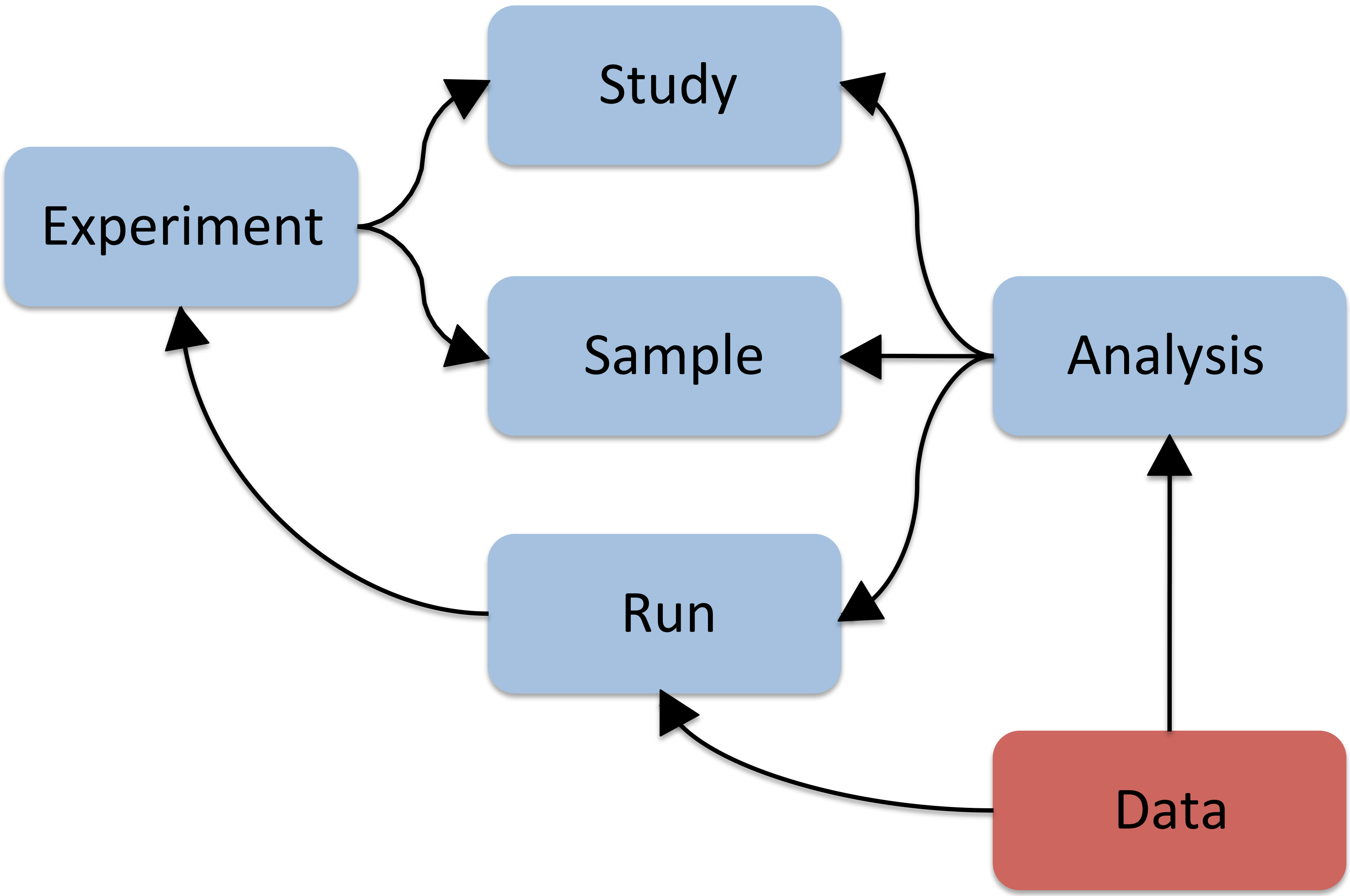

Figure

Click here to download Figure Figure3.pdf

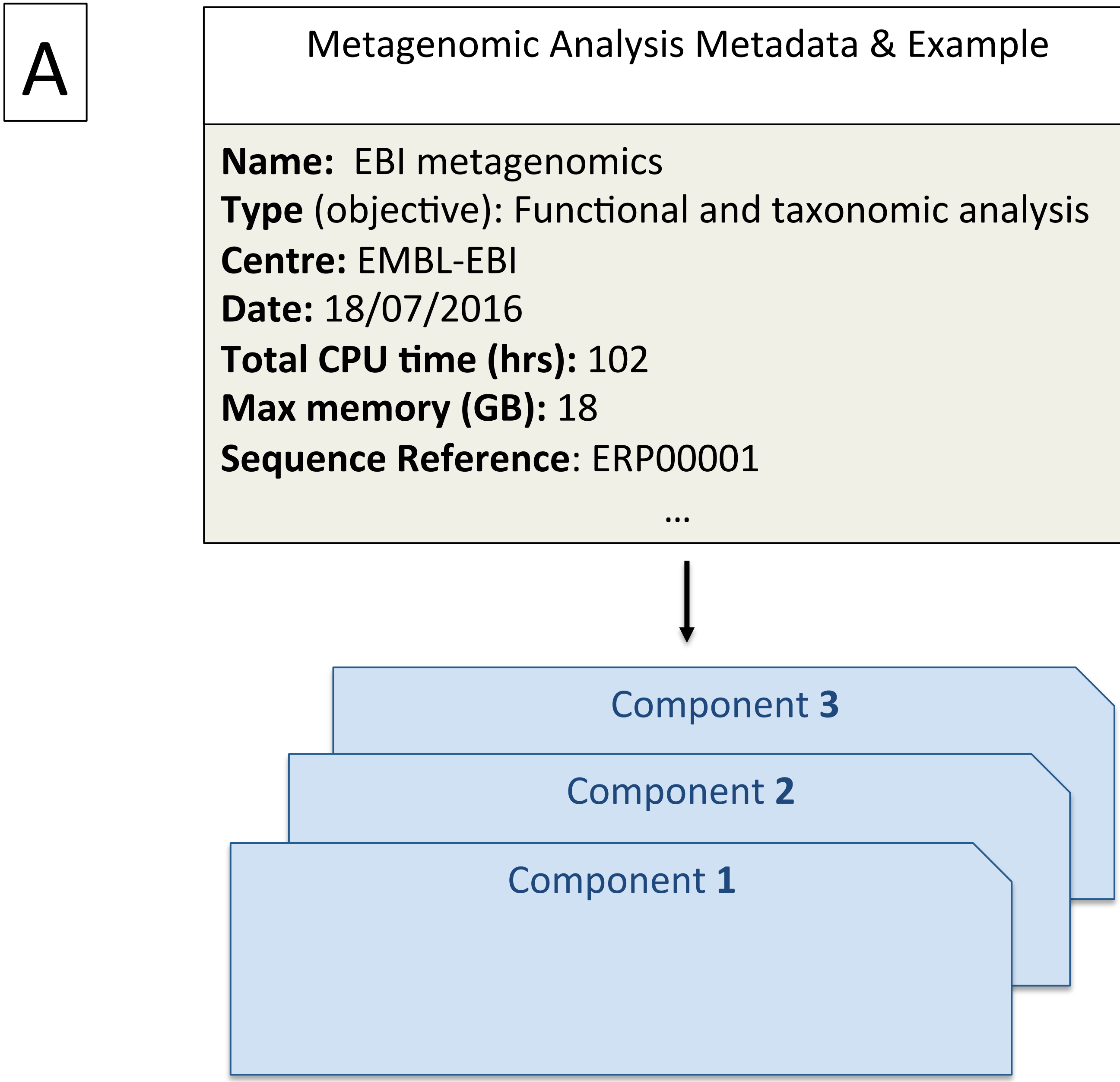

B

Component Metadata & Example

**Name**: InterProScan	
Input(s)	Output 1<br>Component: FragGeneScan
	**Reference DBs**: InterPro 58.0 signature lib.
Tools	**Name**: InterPro
	**Version**: 5.19-58.0
	**Source Code**: https://www.ebi.ac.uk/interpro/interproscan.html
	**Parameters**
Output(s)	1. Hit XML<br>2. GO slim

C

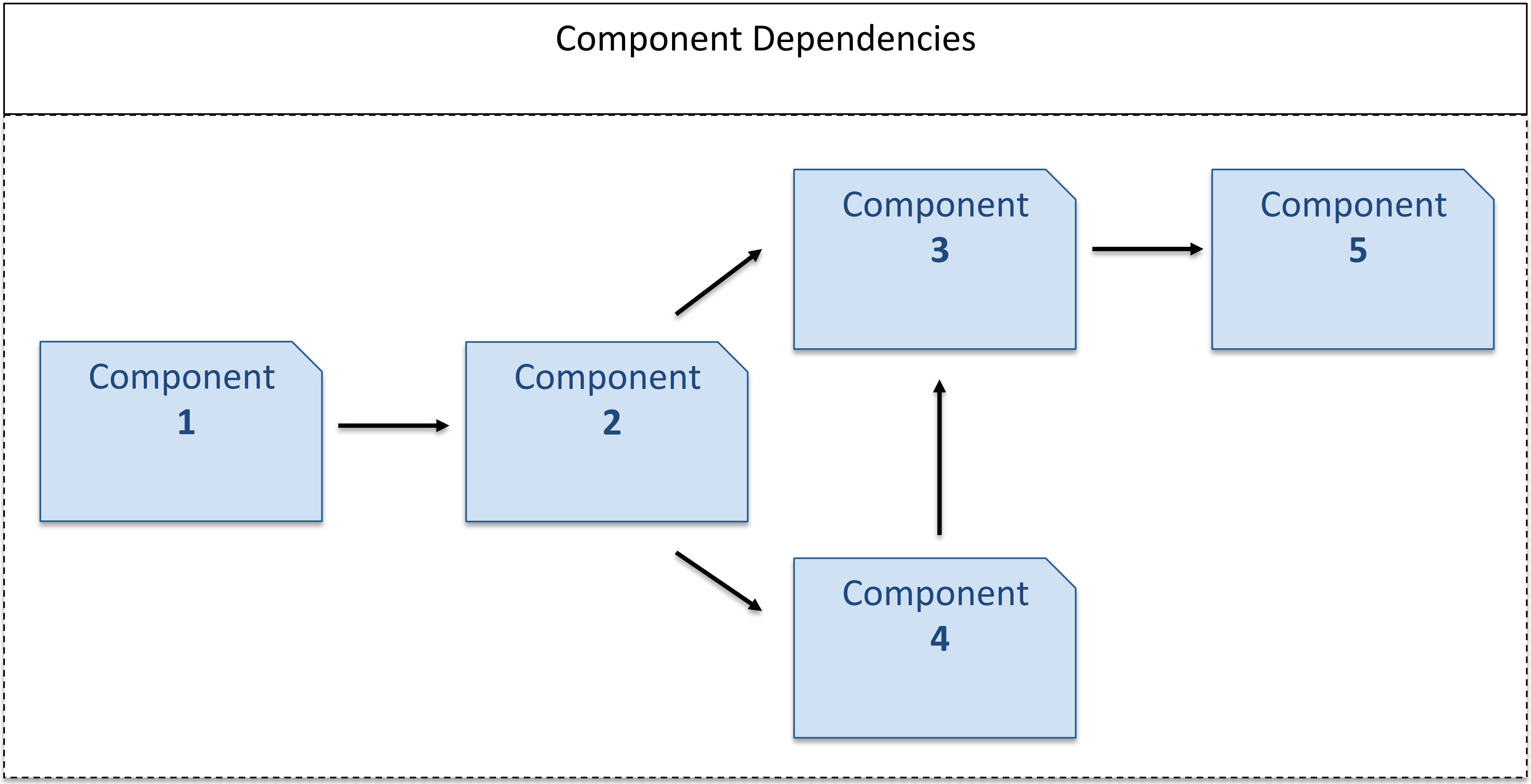

Component Dependencies
Component 1
Component 2
Component 3
Component 4
Component 5
