# Supplementary material for: The metagenomic data life-cycle: standards and best practices
Source: Gigascience. 2017 Jun 16;6(8):1–11. doi: 10.1093/gigascience/gix047 (PMC5737865; doi:10.1093/gigascience/gix047)
Supplement: Response_to_Reviewer_Comments_Original_Submission.pdf [file gix047_response_to_reviewer_comments_original_submission.pdf]

Author's Response To Reviewer Comments

- We thank the reviewers for their work in assessing and helping to improve the manuscript; we insert our comments and responses to the reviewers' points in their text below.

Reviewer #1: GIGA-D-17-00033

This is a timely and well-written article that summarises current or emerging standards for reporting metagenomic projects. A need to harmonise reporting of metagenomic project is certainly given and the manuscript covers all relevant aspects with depth of knowledge. While the main objective of this manuscript is to describe standards for reporting data, it would be also worthwhile mentioning the need (and associated standards) to store and report on the actual biological samples or extracted nucleic acids. These represent very valuable resources both in terms of sampling effort and the fact that they are often unique in time and space. Furthermore those resources could be re-analysed in the future with new sequencing technologies providing additional opportunities to enhance database content. In addition, I have the following comment to consider for improving the manuscript:
- No change; we agree that these are valuable resources, but our focus here is on the data. Capture of this information and the appropriate structures are covered in the specific data standards, so we feel that this is sufficiently covered.

Page 6; Line 54: I think it is important to note that primary data might require additional meta-information. While I appreciate that primary datasets can sometimes already include some basic analysis, information on the type of analysis should be attached to the primary datasets. As primary datasets are sometimes directly compared (e.g. in terms of size or quality) and a lack of information about potential processing would likely lead to bias.
- We introduce a sentence to note this early processing and the importance of describing the applied methods.

Page 12; Line 42: DNA extraction is certainly one step in metagenomics projects, where a large range of protocols exists that would require much detailed information to be captured. However such information can be stored in external databases that specialise in standardising and sharing protocols, such as protocols.io. In fact protocol.io can captured and display variations in protocols, which would facilitate the comparison of studies, and perhaps, in the long run bring users towards using more common extraction methods. I would suggest that the authors consider this as an additional requirement or extension of the desired reporting structure.
- We have added reference to protocols.io as suggested.

Page 15, Line 23: Please provide references for EMG and MG-RAST.
- Not changed; References to EMG and MG-RAST are provided at the first mention of the resources, see page 5.

Reviewer #2: This is an important paper and provides a nice overview of the issues and future directions needed. I have a few minor corrections to suggest (below) and also a general request

to take another look at the overall structure and see if some linker text and a better choice of headings/subheadings might guide the reader through more easily.

Page 4

Line 11: It might be useful here to distinguish metagenomics, metagenetics, and metabarcoding (not to mention amplicon and shotgun metagenomics), as these terms often confused. Or perhaps state that for the purposes of this paper it doesn't matter as they all fall under the "meta - omics" approach and share a similar data model.
- We provide some working definitions for readers of this manuscript.

Page 5

Line 13: The GSC might be added in this section too as a forum/community on which this paper also draws.
- The GSC is mentioned in the following paragraph as a community around standards; we feel that this holds as the mentions at line 13 are ‘research centres of excellence’.

Page 6

Line 15: Study - it might be good to add "including information on any legal issues surrounding the access to the genetic materials and subsequent conditions of use." For example, biodiversity studies should include information on Access and Benefit Sharing (ABS) provisions under the CBD Nagoya Protocol.
- We inserted reference to best scientific practice and ABS compliance under the ‘metadata’ introductory sentence in ‘Overview of the metagenomics data model’.

Line 20: Sample - and information about how/where the sample is stored including long term archiving (i.e., the physical materials).
- Not changed; we provide this information later in the manuscript – specifically in the ‘Sampling’ section; here we are simply introducing the concept of sample in the metagenomics data model.

A general point on the structure of the paper arises here. There is a bullet point of the "objects" representing the five "concepts": Study, Sample, Experiment, Run, Analysis (also in Figure 2). Then there is some discussion on how the authors use the terms metadata, primary data, and derived data. That all seems fine but then in Line 21 there is a discussion related primarily to "Study", which seems like it could be under a subheading "Study"… and the reader might then expect subheadings discussing the other objects/concepts. But after this, there are four headings: Sampling, Sequencing, Analysis, and Archiving, which all make sense but seem poorly introduced.
- We rephrase this to refer to metagenomics ‘initiatives’; the aim here is not to refer to study records in the data model, rather the entirety of a metagenomics effort and the important of tracking people’s activity in this.

Perhaps some additional text is needed here just to make the transition clear and to explain the flow of the paper a bit better.
- We have added a paragraph to explain the flow of the paper at the end of the “Background” section.

Line 51: A general point, but would it not be appropriate to mention the concepts of BioProject and Biosample and how they relate to "Study" and "Sample"?
- We have intentionally avoided using the names “BioProject” and “BioSample”; while it is correct that a user of NCBI or EBI would “register samples with one the BioSamples database at EBI” or “submit studies to BioProject at NCBI”, these are brand names that refer to specific implementations; here we aim to discuss the data model in general brand-neutral terms.

Page 8

Line 26: Geo Bon should be spelled out in full and then acronym capitalized GEO BON. The main reference to GEO BON (7) URL http://geobon.org/essential-biodiversity-variables/connect-with-geoss/ goes nowhere. Better to cite GEO BON website: https://www.earthobservations.org

It might just be me, but saying GEO BON is for "biodiversity data" seems odd as it implies biodiversity does not include genomics data or proteomics data, which were previously assigned to GSC and PSI. Perhaps say:

"…or the Group on Earth Observations Biodiversity Observations Network (GEO BON) for the various dimensions of biodiversity (cite website and perhaps (8)), including genetic variation, then cite the following instead of the current (8): Bruford MW, Davies N, Dulloo ME, Faith DP, Walters M: Monitoring Changes in Genetic Diversity. In The GEO Handbook on Biodiversity Observation Networks. Edited by Walters M, Scholes RJ: Springer; 2017: 107-128
- Edited as suggested; Graziano please check this as I think you contributed the GEO BON text.

Line 30: Again, the transition here is unclear. Having listed GSC, PSI, and GEO BON, the next section seems to start expanding on GSC standards. But then the reader might expect similar for PSI and GEO BON.
- We expanded in the text on the GSC standards as these are the most directly relevant for this paper; accepting the suggestion from the reviewer, we now add text to highlight that we will expand on the GSC standards.

Line 46: metabarcoding is mentioned here (which is fine) but could have been flagged earlier on (see comment above).
- We have added text to introduce and define “metabarcoding” in para. 1 of the “Background” section.

Page 9

Line 53: It is not clear why we jump to M2B3 here, or indeed how it relates to the GSC standards just presented. It sounds somewhat like the NEON/CZO "profiles" mentioned later in the paper.

Some explanation needed.
- See response to comment on Page 8, Line 30 above.

Page 10

Line 44-52: This paragraph is rather obtuse. I, at least, have a hard time understanding what point is being made.
- We have rewitten for clarity.

Page 12

Line 30: just a general point that this reflects a hand-over for Field Information Management Systems (FIMS) to LIMS. See http://fims.readthedocs.io/en/latest/ for an example of FIMS.
- This is a valid point, but we feel for those readers working at a small scale, introducing FIMS and LIMS would add complexity – as these readers won’t be familiar with these systems – so make no change here.
