# Supplementary material for: The metagenomic data life-cycle: standards and best practices
Source: Gigascience. 2017 Jun 16;6(8):1–11. doi: 10.1093/gigascience/gix047 (PMC5737865; doi:10.1093/gigascience/gix047)
Supplement: Reviewer_1_Report_(Original_Submission).pdf [file gix047_reviewer_1_report_(original_submission).pdf]

Reviewer Report

Title: The metagenomic data life-cycle: standards and best practices

Version: Original Submission **Date:** 2/20/2017

Reviewer name: Torsten Thomas

Reviewer Comments to Author:

GIGA-D-17-00033This is a timely and well-written article that summarises current or emerging standards for reporting metagenomic projects. A need to harmonise reporting of metagenomic project is certainly given and the manuscript covers all relevant aspects with depth of knowledge. While the main objective of this manuscript is to describe standards for reporting data, it would be also worthwhile mentioning the need (and associated standards) to store and report on the actual biological samples or extracted nucleic acids. These represent very valuable resources both in terms of sampling effort and the fact that they are often unique in time and space. Furthermore those resources could be re-analysed in the future with new sequencing technologies providing additional opportunities to enhance database content. In addition, I have the following comment to consider for improving the manuscript:Page 6; Line 54: I think it is important to note that primary data might require additional meta-information. While I appreciate that primary datasets can sometimes already include some basic analysis, information on the type of analysis should be attached to the primary datasets. As primary datasets are sometimes directly compared (e.g. in terms of size or quality) and a lack of information about potential processing would likely lead to bias.Page 12; Line 42: DNA extraction is certainly one step in metagenomics projects, where a large range of protocols exists that would require much detailed information to be captured. However such information can be stored in external databases that specialise in standardising and sharing protocols, such as protocols.io. In fact protocol.io can captured and display variations in protocols, which would facilitate the comparison of studies, and perhaps, in the long run bring users towards using more common extraction methods. I would suggest that the authors consider this as an additional requirement or extension of the desired reporting structure.Page 15, Line 23: Please provide references for EMG and MG-RAST.

Level of Interest

Please indicate how interesting you found the manuscript: An article of importance in its field

Quality of Written English

Please indicate the quality of language in the manuscript: Acceptable

Declaration of Competing Interests

Please complete a declaration of competing interests, considering the following questions:

- Have you in the past five years received reimbursements, fees, funding, or salary from an organisation that may in any way gain or lose financially from the publication of this manuscript, either now or in the future?
- Do you hold any stocks or shares in an organisation that may in any way gain or lose financially from the publication of this manuscript, either now or in the future?
- Do you hold or are you currently applying for any patents relating to the content of the manuscript?
- Have you received reimbursements, fees, funding, or salary from an organization that holds or has applied for patents relating to the content of the manuscript?
- Do you have any other financial competing interests?
- Do you have any non-financial competing interests in relation to this paper?

If you can answer no to all of the above, write 'I declare that I have no competing interests' below. If your reply is yes to any, please give details below.

I declare that I have no competing interests.

I agree to the open peer review policy of the journal. I understand that my name will be included on my report to the authors and, if the manuscript is accepted for publication, my named report including any attachments I upload will be posted on the website along with the authors' responses. I agree for my report to be made available under an Open Access Creative Commons CC-BY license (http://creativecommons.org/licenses/by/4.0/). I understand that any comments which I do not wish to be included in my named report can be included as confidential comments to the editors, which will not be published.

I agree to the open peer review policy of the journal
