# Supplementary material for: The metagenomic data life-cycle: standards and best practices
Source: Gigascience. 2017 Jun 16;6(8):1–11. doi: 10.1093/gigascience/gix047 (PMC5737865; doi:10.1093/gigascience/gix047)
Supplement: Reviewer_2_Report_(Original_Submission).pdf [file gix047_reviewer_2_report_(original_submission).pdf]

Reviewer Report

Title: The metagenomic data life-cycle: standards and best practices

Version: Original Submission **Date:** 3/3/2017

Reviewer name: Neil DAVIES

Reviewer Comments to Author:

This is an important paper and provides a nice overview of the issues and future directions needed. I have a few minor corrections to suggest (below) and also a general request to take another look at the overall structure and see if some linker text and a better choice of headings/subheadings might guide the reader through more easily.Page 4Line 11: It might be useful here to distinguish metagenomics, metagenetics, and metabarcoding (not to mention amplicon and shotgun metagenomics), as these terms often confused. Or perhaps state that for the purposes of this paper it doesn't matter as they all fall under the "meta - omics" approach and share a similar data model.Page 5Line 13: The GSC might be added in this section too as a forum/community on which this paper also draws.Page 6Line 15: Study - it might be good to add "including information on any legal issues surrounding the access to the genetic materials and subsequent conditions of use." For example, biodiversity studies should include information on Access and Benefit Sharing (ABS) provisions under the CBD Nagoya Protocol.Line 20: Sample - and information about how/where the sample is stored including long term archiving (i.e., the physical materials).A general point on the structure of the paper arises here. There is a bullet point of the "objects" representing the five "concepts": Study, Sample, Experiment, Run, Analysis (also in Figure 2). Then there is some discussion on how the authors use the terms metadata, primary data, and derived data. That all seems fine but then in Line 21 there is a discussion related primarily to "Study", which seems like it could be under a subheading "Study"… and the reader might then expect subheadings discussing the other objects/concepts. But after this, there are four headings: Sampling, Sequencing, Analysis, and Archiving, which all make sense but seem poorly introduced.Perhaps some additional text is needed here just to make the transition clear and to explain the flow of the paper a bit better.Line 51: A general point, but would it not be appropriate to mention the concepts of BioProject and Biosample and how they relate to "Study" and "Sample"?Page 8Line 26: Geo Bon should be spelled out in full and then acronym capitalized GEO BON. The main reference to GEO BON (7) URL http://geobon.org/essential-biodiversity-variables/connect-with-geoss/ goes nowhere. Better to cite GEO BON website: https://www.earthobservations.orgIt might just be me, but saying GEO BON is for "biodiversity data" seems odd as it implies biodiversity does not include genomics data or proteomics data, which were previously assigned to GSC and PSI. Perhaps say:"…or the Group on Earth Observations Biodiversity Observations Network (GEO BON) for the various dimensions of biodiversity (cite website and perhaps (8)), including genetic variation, then cite the following instead of the current (8): Bruford MW, Davies N, Dulloo ME, Faith DP, Walters M: Monitoring Changes in Genetic Diversity. In The GEO Handbook on Biodiversity Observation Networks. Edited by Walters M, Scholes RJ: Springer; 2017: 107-128Line 30: Again, the transition here is unclear. Having listed GSC, PSI, and GEO BON, the next section seems to start expanding on GSC standards. But then the reader might expect similar for PSI and GEO

BON.Line 46: metabarcoding is mentioned here (which is fine) but could have been flagged earlier on (see comment above).Page 9Line 53: It is not clear why we jump to M2B3 here, or indeed how it relates to the GSC standards just presented. It sounds somewhat like the NEON/CZO "profiles" mentioned later in the paper. Some explanation needed.Page 10Line 44-52: This paragraph is rather obtuse. I, at least, have a hard time understanding what point is being made.Page 12Line 30: just a general point that this reflects a hand-over for Field Information Management Systems (FIMS) to LIMS. See http://fims.readthedocs.io/en/latest/ for an example of FIMS.

Level of Interest

Please indicate how interesting you found the manuscript: An article of importance in its field

Quality of Written English

Please indicate the quality of language in the manuscript: Acceptable

Declaration of Competing Interests

Please complete a declaration of competing interests, considering the following questions:

- Have you in the past five years received reimbursements, fees, funding, or salary from an organisation that may in any way gain or lose financially from the publication of this manuscript, either now or in the future?
- Do you hold any stocks or shares in an organisation that may in any way gain or lose financially from the publication of this manuscript, either now or in the future?
- Do you hold or are you currently applying for any patents relating to the content of the manuscript?
- Have you received reimbursements, fees, funding, or salary from an organization that holds or has applied for patents relating to the content of the manuscript?
- Do you have any other financial competing interests?
- Do you have any non-financial competing interests in relation to this paper?

If you can answer no to all of the above, write 'I declare that I have no competing interests' below. If your reply is yes to any, please give details below.

I declare that I have no competing interests.

I agree to the open peer review policy of the journal. I understand that my name will be included on my report to the authors and, if the manuscript is accepted for publication, my named report including any attachments I upload will be posted on the website along with the authors' responses. I agree for my report to be made available under an Open Access Creative Commons CC-BY license (http://creativecommons.org/licenses/by/4.0/). I understand that any comments which I do not wish to

be included in my named report can be included as confidential comments to the editors, which will not be published.

I agree to the open peer review policy of the journal
